# Beyond monofunctionality: structural perspectives on phenol oxidase side activity in a catalase

**DOI:** 10.1107/S2059798326009848

**Published:** 2026-10-08

**Authors:** Christina Ferousi, Christos Kosinas, Odyssefs Ioannis Pantelakis, Efstratios Nikolaivits, Evangelos Topakas, Maria Dimarogona

**Affiliations:** a Industrial Biotechnology and Biocatalysis Group, Biotechnology Laboratory, School of Chemical Engineering National Technical University of Athens Athens Greece; b Department of Chemistry University of Copenhagen Copenhagen Denmark; c Laboratory of Structural Biology and Biotechnology, Department of Chemical Engineering University of Patras Patras Greece; d Institute of Molecular Biotechnology Graz University of Technology NAWI Graz Graz Austria; Lund University, Sweden

**Keywords:** catalases, catalytic promiscuity, phenol oxidases, X-ray crystallography, catechol

## Abstract

Catalases are ubiquitous enzymes that protect cells from oxidative stress through the disproportionation of hydrogen peroxide. Although oxidase side activities have been reported for a limited number of catalases, their structural basis and physiological relevance remain poorly understood. This study investigates the catalytic promiscuity of *Cl*CPO, an extracellular fungal catalase from the marine fungus *Cladosporium* sp., previously identified as a potential degrader of persistent organic pollutants. Recombinant *Cl*CPO was biochemically characterized and shown to catalyse the oxidation of *ortho*-diphenols to *ortho*-quinones, bearing hydroxy, ethylamine and single and double chlorine substitutions on the benzene ring, in the absence of hydrogen peroxide, while retaining robust catalase activity. On the contrary, it did not exhibit any cresolase activity in either unsubstituted or chlorinated monophenols, nor phenol oxidative activity on hydroxy-biphenyls. Crystal structures of apo *Cl*CPO and a catechol-bound complex were determined at high resolution, identifying a catechol-binding site located at the junction of the main and secondary access channels to the haem cofactor. Docking simulations suggested an additional candidate ligand-binding region capable of accommodating bulkier compounds, whereas *in silico* electron/hole-transfer pathway analysis identified recurrent predicted pathway elements connecting the different ligand-binding regions to the haem centre. Structural comparison with previously characterized bifunctional catalases highlights distinct substrate-recognition features and suggests an alternative mechanistic basis for phenol oxidase activity in *Cl*CPO. Together, these findings provide structural insight into the catalytic promiscuity of *Cl*CPO, support a working mechanistic framework for phenol oxidase activity in fungal catalases and expand our understanding of the functional diversity of haem-dependent oxidoreductases.

## Abstract 2

Structural and biochemical characterization of the extracellular fungal catalase *Cl*CPO uncovers an unexpected phenol oxidase promiscuity. Crystal structures, substrate docking and electron-transfer pathway analysis identify ligand-binding regions and support a working mechanistic model for catalase-associated phenol oxidase activity.

## 1. Introduction

Catalases are enzymes located either in the peroxisome of eukaryotes or the cytosol of prokaryotes, and their main role is the scavenging of hydrogen peroxide (H_2_O_2_), reducing oxidative stress within the cell. Most typical catalases are homotetramers with four haem prosthetic groups. Each monomer is composed of four distinct domains: (i) the N-terminal arm, which participates in an inter-subunit knot-like connection, (ii) the β-barrel domain, which contains the haem and is the most conserved catalase domain, (iii) the ‘wrapping domain’, which also mediates oligomerization, and (iv) the α-helical domain (Zámocký & Koller, 1999 ▸).

In addition to dismutation of hydrogen peroxide, some catalases also display peroxidase and oxidase activities. Thus, depending on their physical and biochemical properties, they are divided into monofunctional haem catalases, catalase–peroxidases, nonhaem catalases (pseudocatalases) and minor catalases (Sooch *et al.*, 2014 ▸). The first step of the catalatic mechanism is the reduction of one molecule of H_2_O_2_ to H_2_O, upon which the haem Fe^3+^ is converted to Fe^4+^=O (Compound I). In cases where Compound I does not oxidize H_2_O_2_ to molecular oxygen and water (low H_2_O_2_ concentration), catalases act as peroxidases, oxidizing alcohols (Nicholls, 2012 ▸). On the other hand, minor catalases comprise bifunctional haem enzymes that are able to also act as phenol oxidases, independently of the presence of H_2_O_2_, and are named catalase–phenol oxidases (CATPOs). Oxidase activity in catalases can exhibit broad substrate specificity, involving structurally diverse compounds such as dichlorofluorescein (DCFH) and the orthodiphenols catechol and pyrogallol, as well as caffeic acid and l-DOPA (Sutay Kocabas *et al.*, 2008 ▸; Vetrano *et al.*, 2005 ▸). Phenol oxidase activity was first demonstrated in mammalian catalase. This activity was independent of the enzyme’s catalatic and peroxidatic functions and displayed promiscuity, oxidizing a broad range of substrates. A putative binding site was proposed in the vicinity of the β-barrel region and was suggested to involve a cluster of conserved Arg residues (Vetrano *et al.*, 2005 ▸). One of the best-studied catalases with phenol oxidase activity is CATPO from *Scytalidium thermophilum* (*St*CATPO). The phenol oxidase activity of *St*CATPO is independent of H_2_O_2_, and its preferred substrate is catechol (Sutay Kocabas *et al.*, 2008 ▸). The haem group acts as a redox centre for both catalatic and oxidative activities; however, binding of phenolic substrates was suggested to occur at a site linked to the haem via an electron-transfer pathway (Yuzugullu *et al.*, 2013 ▸). This was corroborated by the crystallographic structure of *St*CATPO in complex with the widely used catalase inhibitor 3-amino-1,2,4-triazole (3TR; Yuzugullu Karakus *et al.*, 2018 ▸). The identified oxidase-substrate binding pocket differs from the site proposed for mammalian catalase; it is situated at the entrance of a lateral channel linking the haem to the molecular exterior, a region occupied by NADPH in mammalian catalase. The entrance to the lateral pocket as a putative binding site for oxidase substrates was further supported through mutational and kinetic studies (Goc *et al.*, 2021 ▸). Although additional catalase–phenol oxidases have been biochemically studied (Teng *et al.*, 2016 ▸; Sangar *et al.*, 2012 ▸; Lončar & Fraaije, 2015 ▸), to our knowledge there is no other crystallographically determined complex of a CATPO, corroborating the binding site of phenolic compounds that are subsequently oxidized.

Here, we investigate the catalytic promiscuity of an extracellular fungal catalase/phenol oxidase from the marine fungus *Cladosporium* sp., termed *Cl*CPO. *Cladosporium* sp. was previously identified as a potential degrader of polychlorinated biphenyls (PCBs), persistent organic pollutants with severe environmental and health impacts. Transcriptomic analysis revealed the upregulation of several genes encoding extracellular oxidoreductases with putative roles in pollutant degradation, including the gene encoding *Cl*CPO (Nikolaivits *et al.*, 2021 ▸). Given its extracellular localization, *Cl*CPO is unlikely to function solely in detoxification of reactive oxygen species, suggesting a possible role in extracellular oxidative transformations associated with pollutant degradation. However, whether *Cl*CPO directly participates in PCB transformation has not been experimentally established. Here, we present a detailed biochemical and structural characterization of recombinant *Cl*CPO, including its crystal structure in the presence of catechol, and investigate candidate ligand-binding regions that may contribute to the observed phenol oxidase side activity.

## 2. Experimental procedures

### 2.1. Materials

Unless otherwise stated, all chemicals were obtained from commercial suppliers at the highest available purity and were used without further purification. *Escherichia coli* TOP10 and *Komagataella phaffii* X33 Mut+ strains were purchased from Thermo Fisher Scientific (USA). The recombinant pPICZαC/*Cl*CPO plasmid vector was synthesized by GenScript Biotech B.V. (Netherlands). Zeocin was purchased from InvivoGen (USA). NucleoSpin plasmid kits were supplied by Macherey-Nagel (Germany). PmeI and EndoH were obtained from New England Biolabs (USA). TALON Superflow resin for metal-affinity chromatography was purchased from Merck (Germany). Catalase from bovine liver (CAS 9001-05-2) was purchased from Merck (Germany). Tyrosinase-like polyphenol oxidase from *Thermothelomyces thermophila* (*Tt*PPO; UniProt ID G2QLD3) was recombinantly expressed in *K. phaffii* and purified as described previously (Nikolaivits *et al.*, 2018 ▸). Other chemicals, including phenolic compounds, were purchased from Sigma–Aldrich (USA).

### 2.2. Cloning, protein expression and purification

The nucleotide sequence encoding *Cl*CPO (excluding the native signal sequence) was derived from previously reported data (Nikolaivits *et al.*, 2018 ▸), codon-optimized for expression in *K. phaffii*, synthesized and cloned into the pPICZαC vector, operating under the control of the PAOX1 promoter and containing zeocin resistance, in frame with the α-factor secretion signal at the N-terminus, as well as both a c-Myc epitope and a polyhistidine (His)_6_ tag at the C-terminus. For plasmid amplification, constructs were transformed into chemically competent *E. coli* TOP10 cells through heat-shock, transformants were grown on Luria–Bertani (LB) medium supplemented with 25 µg ml^−1^ zeocin at 37°C for 16 h and plasmid extraction was performed with the NucleoSpin Plasmid kit. Follow-up preparation of the recombinant *K. phaffii* strain was performed according to the EasySelect Pichia Expression Kit (Thermo Fisher Scientific, USA). The obtained plasmids were linearized with PmeI, transformed into competent *K. phaffii* cells via electroporation and subsequently grown on yeast peptone dextrose sorbitol (YPDS) plates supplemented with 100 µg ml^−1^ zeocin at 30°C for three days. Precultures of zeocin-resistant selected strains were prepared in buffered glycerol-complex medium (BMGY) and incubated at 28°C for 24 h. Methylotrophic cultivation of recombinant *K. phaffii* was performed in 500 ml buffered methanol-complex medium (BMMY) with a headspace-to-liquid volume ratio of 4:1 at 20°C in shaken flasks with the addition of 0.5%(*v*/*v*) methanol daily for five days. The medium was supplemented with 10 µ*M* hemin and 4 ml l^−1^ Trace Elements Solution (TES; composition: 3.83 g l^−1^ CuSO_4_·5H_2_O, 0.08 g l^−1^ NaI, 3.36 g l^−1^ MnSO_4_·H_2_O, 0.18 g l^−1^ Na_2_MoO_4_·2H_2_O, 0.02 g l^−1^ H_3_BO_3_, 1.36 g l^−1^ ZnCl_2_, 2.78 g l^−1^ FeSO_4_·7H_2_O, 0.50 g l^−1^ CoCl_2_·6H_2_O, 1 ml l^−1^ 98% H_2_SO_4_).

The culture supernatant was harvested, filtered through 0.22 µm polyethersulfone (PES) membrane filters (Membrane Solutions, USA), concentrated 20-fold via a 10 kDa cutoff PES ultrafiltration membrane (Amicon Stirred Cell; Merck Millipore, Germany) and subjected to haem reconstitution at 4°C overnight, with the addition of hemin at a fivefold molar excess to total protein (MW = 65 000 g mol^−1^ was assumed for molarity calculations). A 2 m*M* hemin stock solution was prepared in dimethyl sulfoxide (DMSO) and the latter was always present at less than 5%(*v*/*v*) in all protein samples. *Cl*CPO was purified via gravity-flow immobilized metal-affinity chromatography equilibrated with 20 m*M* Tris–HCl buffer containing 0.3 *M* NaCl pH 8.0. After sample application, the column was first washed with ten column volumes (CV) of 20 m*M* Tris–HCl buffer pH 8.0 containing 0.3 *M* NaCl, and then with 3 CV of 5 m*M* imidazole in the same buffer. *Cl*CPO eluted isocratically with 2 CV of 0.1 *M* imidazole in the same buffer and these two fractions were routinely collected separately for purity and haem-content evaluation via the Reinheitszahl (Rz) ratio. *N*-Deglycosylation of denatured *Cl*CPO was performed with 20-fold diluted Endo H according to the manufacturer’s protocol and incubation at 4°C overnight. Sample purity was checked by SDS–denaturing polyacrylamide gel electrophoresis (Laemmli, 1970 ▸). Protein concentration was determined either by the Bradford assay (Bio-Rad, USA) with bovine serum albumin as standard (Bradford, 1976 ▸) or by measuring the absorbance at 280 nm. The *ProtParam* tool from ExPASy was utilized for computation of the theoretical pI, MW and molar extinction coefficient (Gasteiger *et al.*, 2005 ▸). *N*- and *O*-glycosylation sites were predicted by the *NetNGlyc* and *NetOGlyc* online tools, respectively (Gupta & Brunak, 2002 ▸; Steentoft *et al.*, 2013 ▸).

### 2.3. Biochemical characterization

UV–visible spectra were recorded at room temperature (RT) in 1.5 ml quartz cuvettes (path length 1 cm; Hellma) using a spectrophotometer (Shimadzu UV-1280, Japan) operated via the *SoftMaxPro* software (version 1.1, Molecular Devices, Sunnyvale, California, USA). Enzymatic activity was expressed in units (U), representing the quantity of enzyme capable of consuming or producing 1 µmol substrate or product, respectively, per minute. Catalase activity was measured via two methods. The one-step assay was performed in 1.5 ml quartz cuvettes (path length 1 cm; Hellma) at RT and consisted of 0–25 m*M* H_2_O_2_ and 1–3 n*MCl*CPO in 50 m*M* potassium phosphate buffer pH 8.0. Decomposition of H_2_O_2_ was monitored by the decrease in absorbance at 240 nm (ɛ_240_ = 39.4 *M*^−1^ cm^−1^; Nelson & Kiesow, 1972 ▸) for 2 min. Initial reaction rates were calculated from the linear portion of the absorbance traces during the first 20 s following reaction initiation. The coupled assay was adapted from Hadwan & Ali (2018 ▸) with modifications. After enzymatic H_2_O_2_ decomposition, the remaining H_2_O_2_ was allowed to react with ammonium metavanadate, leading to the formation of a red/orange peroxo vanadium complex:
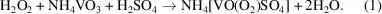


Reactions were performed in 96-well microtiter plates by mixing 80 µl 10 m*M* H_2_O_2_ (6.7 m*M* final concentration) with 40 µl enzyme solution (1–3 n*M* final concentration) in 50 m*M* of an appropriate buffer and allowing the reaction to proceed at RT for 2 min. Quenching with 80 µl 10 m*M* ammonium metavanadate in 0.5 *M* sulfuric acid was followed by absorbance reading at 452 nm using a SpectraMax-250 microplate reader (Molecular Devices, Sunnyvale, California, USA) operated via the *SoftMaxPro* software (version 1.1; Molecular Devices, Sunnyvale, California, USA). Determination of the enzyme activity was based on freshly prepared H_2_O_2_ calibrants.

The catalase pH optimum (pH_opt_) was determined via the coupled assay performed in the pH range 4.0–11.0 with 1.2 n*MCl*CPO at RT in 50 m*M* buffer (pH 4.0–5.0, sodium acetate/acetic acid; pH 4.0–6.0, citric acid/potassium phosphate; pH 6.0–7.0, MES; pH 7.0–8.0, HEPES; pH 8.0–9.0, tricine; pH 9.0–11.0, glycine/NaOH). The catalase temperature optimum (*T*_opt_) was determined via the coupled assay performed in the range 10–80°C with 1.2 n*MCl*CPO in 50 m*M* HEPES pH 8.0, while the catalytic thermal stability was determined by assaying the residual relative activity after incubation at 10–80°C for 0.1–6 h with 2.5 n*MCl*CPO in 50 m*M* HEPES pH 8.0. The peroxidase assay was performed in 50 m*M* citrate–phosphate buffer pH 4.5 containing 2 m*M* 2,2′-azino-bis(3-ethylbenzthiazoline-6-sulfonic acid) (ABTS) and 5 µ*M* H_2_O_2_ at 35°C in 96-well microtiter plates. Reactions were initiated by the addition of 1.9 µ*MCl*CPO and ABTS oxidation was followed at 420 nm (ɛ_420_ = 36 000 *M*^−1^ cm^−1^; Bourbonnais *et al.*, 1998 ▸) for 30 min. The laccase assay followed the same setup as the peroxidase assay with the omission of H_2_O_2_.

The phenol oxidase activity of *Cl*CPO was spectrophoto­metrically evaluated against a selected panel of mono- and biphenolic compounds chosen to sample differences in hydroxylation pattern, aromatic substitution and molecular size, namely phenol, 2-chlorophenol, 4-chlorophenol, 2,5-dichlorophenol, 3,5-dichlorophenol, benzene-1,2-diol (catechol; CAQ), benzene-1,2,3-triol (pyrogallol), (*S*)-2-amino-3-(3,4-dihydroxyphenyl)propanoic acid (l-DOPA), 4-[(1*R*)-1-hydroxy-2-(methylamino)ethyl]benzene-1,2-diol (epinephrine), 4-chlorobenzene-1,2-diol (4-chlorocatechol; 4CH), 3,5-di­chlorobenzene-1,2-diol (3,5-dichlorocatechol; 35D), (*E*)-3-(3,4-dihydroxyphenyl)prop-2-enoic acid (caffeic acid), (2*R*,3*S*)-2-(3,4-dihydroxyphenyl)-3,4-dihydro-2H-chromene-3,5,7-triol (catechin), 2-phenylphenol (2-hydroxybiphenyl), 3-phenylphenol (3-hydroxybiphenyl) and 3-phenylbenzene-1,2-diol (2,3-dihydroxybiphenyl; 2HY). For comparison, commercial bovine liver catalase (BLC) and recombinant polyphenol oxidase (*Tt*PPO) were tested against a representative subset of the same substrates. The assays were performed in 0.1 *M* potassium phosphate buffer pH 7.0 with 2 m*M* substrate at 35°C for 24 h, and the reactions were initiated by the addition of 1.5–2.5 µ*M* enzyme. UV–visible spectra (350–700 nm) of the reaction mixtures were recorded via a SpectraMax-250 microplate reader (Molecular Devices, Sunnyvale, California, USA) at defined time points and were compared with the corresponding controls. Product formation was typically associated with absorption maxima in the range 400–480 nm. Precipitation was frequently observed during the assays, possibly due to oligomerization of oxidized quinone products, which precluded quantitative analysis. Activity was therefore assessed by visual inspection and is reported qualitatively as positive or negative in Fig. 2.

Catecholase activity of *Cl*CPO (1.5 µ*M*) was also evaluated in 0.1 *M* potassium phosphate buffer pH 7.0 containing 0–0.85 *M* catechol at 35°C. Catechol oxidation was followed by the decrease in absorbance at 420 nm (ɛ_420_ = 3450 *M*^−1^ cm^−1^; Ögel *et al.*, 2006 ▸) during the first 2 min of the reaction.

Enzymatic reactions with 1,2,4-trichloro-5-phenylbenzene (PCB29) were performed in 100 m*M* potassium phosphate buffer pH 7.0 with 1 µ*M* PCB29, 1 µ*M* ABTS as a redox mediator and 5 µ*MCl*CPO at 35°C for 24 h. Following incubation, PCB29 was extracted with an equal volume of ethyl acetate at 10°C for 18 h under agitation. The resulting organic phase was analysed by gas chromatography coupled to electron-capture detection (GC-ECD) as described previously (Nikolaivits *et al.*, 2021 ▸).

### 2.4. Protein crystallization

*Cl*CPO was subjected to crystallization using the vapour-diffusion method with the commercially available Morpheus screening kit (Gorrec, 2009 ▸). Protein concentrated to 12.5 mg ml^−1^ was mixed in equal volumes with the crystallization solution. The crystallization setup was prepared using an Oryx Nano crystallization robot. *Cl*CPO crystals appeared in several wells after a few weeks of incubation at 20°C. Crystals grown under the E5 and F9 crystallization conditions were also soaked for several minutes in mother-liquor solutions, each supplemented with a single *Cl*CPO ligand, *i.e.* CAQ, 4CH, 35D and 2HY. Ligands were initially dissolved in DMSO and then diluted with the mother liquor to a final concentration of 50 m*M*. After soaking, the crystals were harvested for data collection. *Cl*CPO (12.5 mg ml^−1^) was also submitted to co-crystallization with 170 m*M* 2HY. The solution was incubated for 30 min at room temperature before being subjected to random microseed matrix screening (rMMS), using seed stock derived from crystals in the F1 well of the Morpheus screen. Protein crystals originating from condition H1 of Morpheus were selected for data collection. The Morpheus crystallization conditions are described in Gorrec (2009 ▸).

### 2.5. Data collection, structure determination and refinement

Crystals obtained from soaking and co-crystallization were harvested and shipped to the P13 beamline at the PETRA III storage ring (DESY, Hamburg, Germany) for data collection (Cianci *et al.*, 2017 ▸). A dataset collected from the co-crystallization setup with 2HY was further processed and led to the determination of the apo structure of the enzyme (apo *Cl*CPO), since the ligand could not be modelled in the electron-density map. Data reduction was performed with *XDS* (Kabsch, 2010 ▸); *AIMLESS* was used for data scaling and cutoff, as well as space-group estimation (Evans & Murshudov, 2013 ▸). The apo *Cl*CPO structure was solved using molecular replacement via *MOLREP* (Vagin & Teplyakov, 1997 ▸) as provided in the *CCP*4 crystallographic suite (Agirre *et al.*, 2023 ▸). The crystal structure of *St*CATPO was used for the construction of the starting model (PDB entry 4aum; Yuzugullu *et al.*, 2013 ▸). Refinement and addition of ordered water molecules were performed with *REFMAC*5 (Murshudov *et al.*, 2011 ▸). Nonwater solvent molecules and ligands were modelled via *WinCoot* (Emsley *et al.*, 2010 ▸). Model validation was performed via *MolProbity* (Williams *et al.*, 2018 ▸). The structure has been deposited in the PDB with accession code 9hxt. Another dataset collected from a crystal that was soaked with CAQ for 2 min was further processed to determine the catechol-bound structure (CAQ–*Cl*CPO). Data for CAQ–*Cl*CPO were processed to a final resolution of 1.95 Å. Although the statistics in the outer resolution shell fall below commonly accepted thresholds, this cutoff was selected as it led to a better resolved electron-density map for CAQ modelling, consistent with previous studies that indicate the role of weak high-resolution reflections in map improvement (Karplus & Diederichs, 2015 ▸). Each step of data analysis was performed using the *CCP*4 suite as mentioned above for the apo *Cl*CPO structure (Agirre *et al.*, 2023 ▸). The structure has been deposited in the PDB with accession code 9rwy. X-ray data-collection and refinement statistics for both structures are presented in Table 1 ▸.

### 2.6. Docking simulations

Docking simulations were performed in *YASARA Structure* (Krieger & Vriend, 2014 ▸). The ligand structures were designed using *Lidia* as implemented in *WinCoot* (Emsley *et al.*, 2010 ▸). Ligand structures were cleaned and their hydrogen-bonding network was optimized. Their geometries were also optimized using semi-empirical quantum-mechanics calculations. All ligand structures were also curated with *AutoSMILES*(Jakalian *et al.*, 2002 ▸). The CAQ–*Cl*CPO structure (PDB entry 9rwy) was used as the docking receptor after removing all heteroatoms, ligands and waters. Polar H atoms were added to the *Cl*CPO structure, and the hydrogen network was optimized before proceeding with docking simulations. Global docking in a 103 Å simulation cell defined around the catechol ligand was performed employing *AutoDock Vina*, completing 25 runs per simulation using the default settings (Eberhardt *et al.*, 2021 ▸). The results were clustered and evaluated based on binding energies, dissociation constants and ligand orientation. Ligand-docking poses away from the main channel were rejected. Docking poses are discussed in terms of binding energy scores and calculated *K*_d_.

### 2.7. Electron- or hole-transfer channel prediction

Possible electron- or hole-transfer (EHT) channels in protein–ligand complexes were analysed using the *eMap* 2.0 web application (Boston University; Tazhigulov *et al.*, 2019 ▸). For CAQ, the crystallographically resolved structure was used, whereas for the other ligands (4CH, 35D, 2HY and PCB) the docking results were utilized. Calculations were performed on chain *A* of all protein structures. All aromatic and non-aromatic amino acids were included as potential transfer sites, with pairwise distances computed based on the centre of mass. Surface-exposed residues were defined according to residue depth, and edge weights were assigned using the *eMap* default distance-dependent penalty function. Shortest transfer pathways were identified using the algorithms implemented in *eMap* and ranked according to the cumulative *eMap* score, a penalty-based measure in which smaller values correspond to more favourable pathways. Advanced parameters were kept at their default settings.

## 3. Results and discussion

### 3.1. Production and initial characterization of *Cl*CPO

Recombinant *Cl*CPO was expressed in *K. phaffii* and purified to homogeneity, yielding about 1 mg per litre of culture with an Rz ratio (*A*_406 nm_/*A*_276 nm_) consistently measured at 0.8–1.0, higher than average for typical monofunctional catalases (Fu *et al.*, 2014 ▸). Under standard denaturing conditions, *Cl*CPO appeared as a smear on SDS–PAGE, indicative of high glycosylation. Treatment with endoglycosidase H resolved a single band at 65 kDa, close to the theoretical MW of 62.5 kDa (Supplementary Fig. S1). The UV–visible absorption spectrum of as-isolated *Cl*CPO exhibited features of typical haem-Fe^3+^ catalases, with a Soret maximum at 406 nm and less prominent Q bands at 505 and 540 nm, together with a porphyrin-to-metal charge-transfer band at ∼630 nm, indicative of a pentacoordinated haem (Supplementary Fig. S2; Jakopitsch *et al.*, 2005 ▸). Addition of ascorbic acid or sodium dithionite did not induce any spectral changes, indicative of haem-Fe^3+^ not being readily reducible under the applied conditions and consistent with previous reports (Kobayashi *et al.*, 2006 ▸).

### 3.2. Catalase activity

**Figure 1:**
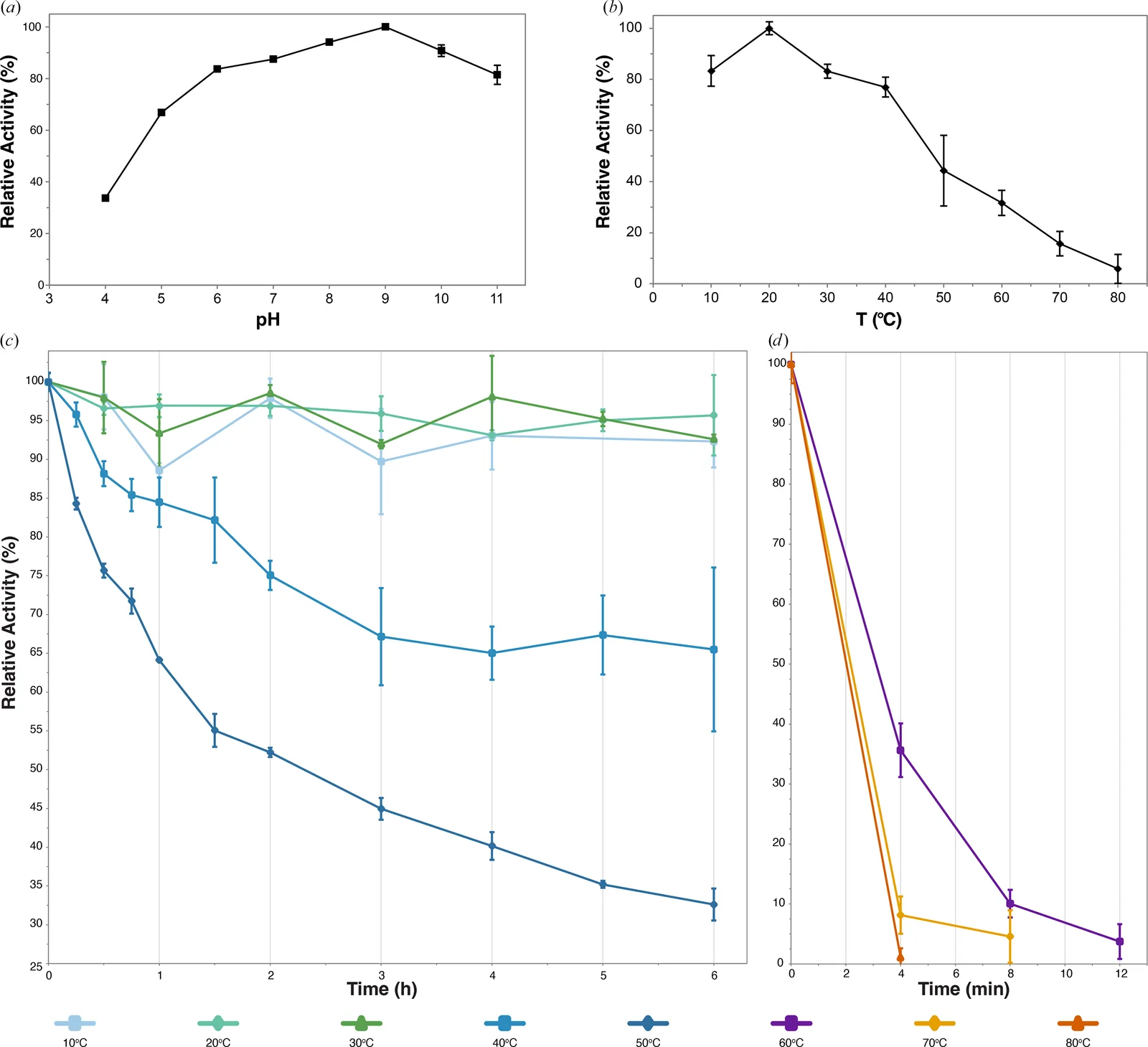
Effect of pH (*a*) and temperature (*b*) on the catalase activity of *Cl*CPO (1.2 n*M*) and the thermal stability of *Cl*CPO (2.5 n*M*) (*c*, *d*). Reactions were performed at RT and rates were quantified using the coupled assay. Activities are expressed as relative activity (%) normalized to the highest observed rate. Values represent the mean of three measurements; error bars indicate standard deviation.

The catalase activity of *Cl*CPO (1.5 n*M*) on 0–25 m*M* H_2_O_2_ was tested both by monitoring the decrease in absorbance at 240 nm and by measuring the formation of a peroxo vanadium complex upon reduction of metavanadate by residual H_2_O_2_ in a coupled assay. In both cases, *Cl*CPO exhibited nonsaturable behaviour, consistent with typical monofunctional catalases that do not follow Michaelis–Menten kinetics (Switala & Loewen, 2002 ▸); the results obtained with the directly monitored assay are shown in Supplementary Fig. S3. The effect of pH on the *Cl*CPO catalatic activity was determined using the coupled assay (1.5 n*MCl*CPO) between pH 4.0 and 11.0 at 25°C. Maximal activity was observed at an apparent pH_opt_ of 9.0, with more than 80% of activity retained between pH 6.0 and 11.0 (Fig. 1 ▸*a*), indicating a comparatively broad pH tolerance among characterized catalases, most of which reach their maximum around pH 7.0–8.0 and typically retain about 50–60% of their activity in the pH range 4.0–12.0. Temperature dependence was assessed in 50 m*M* HEPES buffer pH 8.0. *Cl*CPO exhibited an apparent *T*_opt_ of 20°C and retained less than 40% of its maximal activity above 50°C (Fig. 1 ▸*b*), placing it among mesophilic to psychrophilic counterparts and consistent with its marine origin, as most non-thermotolerant homologues have optimum reaction temperatures between 10 and 40°C and suffer severe inactivation at higher temperatures (Yumoto *et al.*, 2000 ▸; Lorentzen *et al.*, 2006 ▸; Phucharoen *et al.*, 2002 ▸; Wang *et al.*, 2008 ▸). The functional thermostability of *Cl*CPO was evaluated by monitoring the residual catalase activity following prolonged incubation at different temperatures using the coupled assay in 50 m*M* HEPES buffer pH 8.0. In agreement with the *T*_opt_ experiment, *Cl*CPO retained more than 90% of its initial activity after 6 h of incubation between 10 and 30°C, whereas activity decreased to approximately 70% at 40°C and 35% at 50°C (Figs. 1 ▸*c* and 1 ▸*d*). Consistent with other mesophilic to psychrophilic catalases (Yumoto *et al.*, 2000 ▸; Lorentzen *et al.*, 2006 ▸; Phucharoen *et al.*, 2002 ▸; Wang *et al.*, 2008 ▸), *Cl*CPO was rapidly inactivated at temperatures above 60°C, losing activity within 20 min of incubation. After prolonged incubation (three days) at temperatures between 10 and 30°C, enzyme samples retained approximately 90% of their initial activity, whereas samples incubated at 40°C retained approximately 50% (data not shown), contrasting with thermotolerant enzyme homologues (Wang *et al.*, 1998 ▸), despite the overall high sequence conservation.

### 3.3. Peroxidase and phenol oxidase activities

Evaluation of the peroxidatic activity of *Cl*CPO, following the oxidation of ABTS in the presence of minute amounts of H_2_O_2_ (5 µ*M*), further confirmed its classification as a monofunctional haem catalase, as the negligible specific activity observed (4.2 mU mg^−1^) was also obtained in the absence of H_2_O_2_ (*i.e.* laccase activity), indicative of molecular O_2_ being the electron acceptor in the observed redox reaction.

**Figure 2:**
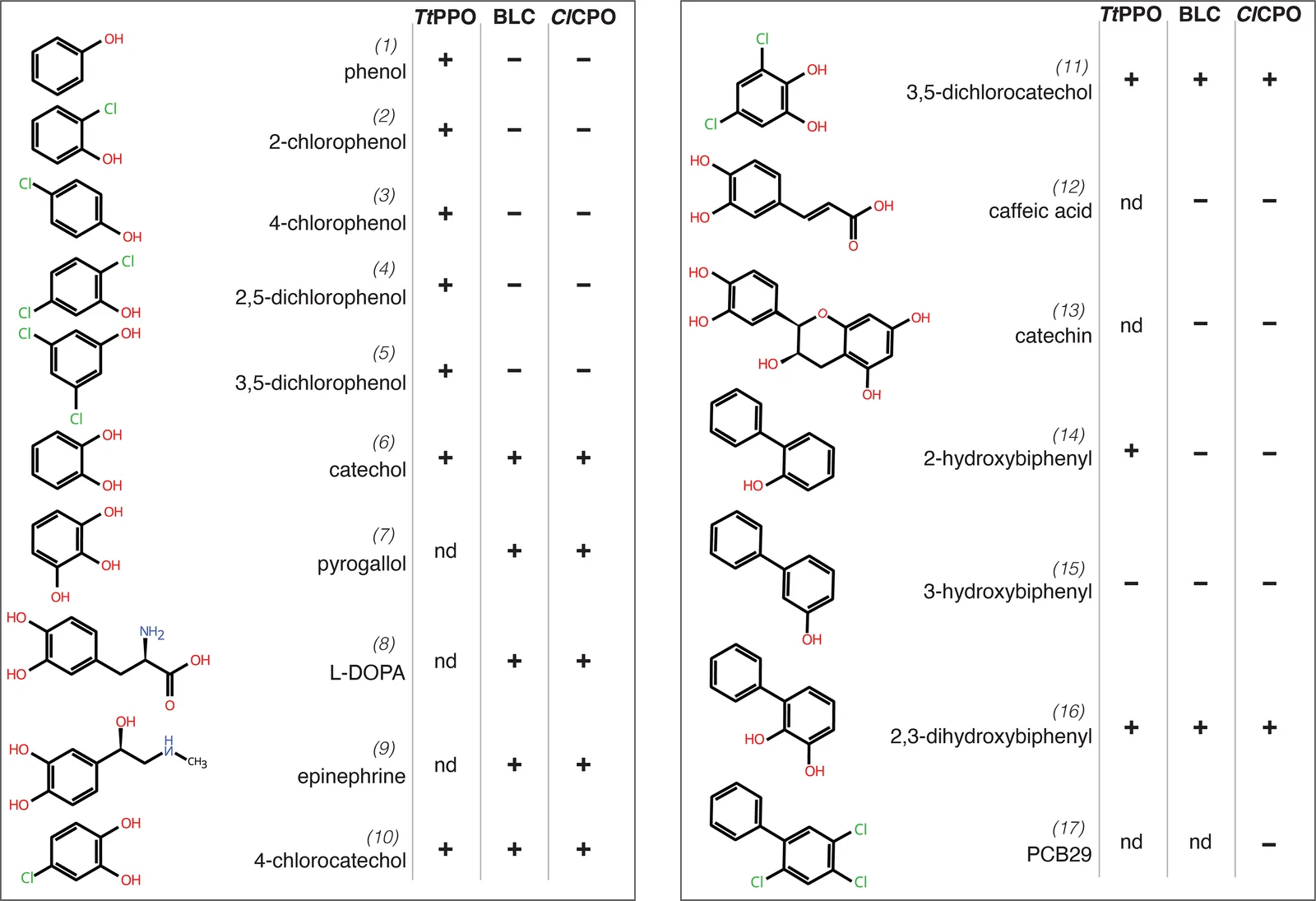
Selected phenolic compounds screened for phenol oxidase activity. The two-dimensional structures and names of the phenolic compounds tested are shown, with compound numbers indicated in parentheses. Phenol oxidase activity of *Cl*CPO, bovine liver catalase (BLC) and *Thermothelomyces thermophila* polyphenol oxidase (*Tt*PPO) towards each compound is indicated as ‘+’ (detectable activity) or ‘−’ (no detectable activity). ND denotes conditions that were not determined.

Given the reported phenol oxidase activity of several catalases (Vetrano *et al.*, 2005 ▸; Teng *et al.*, 2016 ▸; Yuzugullu *et al.*, 2013 ▸), *Cl*CPO was screened against a selected panel of mono- and biphenolic compounds differing in hydroxylation pattern, substitution and molecular size (Fig. 2 ▸). For comparison, BLC and *Tt*PPO (Nikolaivits *et al.*, 2018 ▸) were tested against a representative subset of the same substrates. Neither *Cl*CPO nor BLC exhibited any cresolase activity, *i.e.ortho*-hydroxylation of monophenols to *ortho*-diphenols, in either unsubstituted (Fig. 2 ▸; substrate 1) or chlorinated monophenols (Fig. 2 ▸; substrates 2–5). Oxidation of hydroxy-biphenyls was also not observed (Fig. 2 ▸; substates 14–15). A small decrease in PCB29 concentration was observed in reactions containing *Cl*CPO, both in the absence and presence of ABTS (Supplementary Fig. S4). However, statistical analysis showed that these differences were not significant relative to the corresponding controls. In addition, no identifiable PCB29 transformation products were detected. The present data therefore do not provide sufficient evidence to conclude that *Cl*CPO catalyses PCB29 transformation, and the observed differences may instead reflect experimental variability, non­specific substrate binding or sequestration.

Both *Cl*CPO and BLC exhibited catecholase activity (Fig. 2 ▸; substrate 6), corresponding to the oxidation of *ortho*-diphenols to *ortho*-quinones bearing hydroxy substitutions on the benzene ring (Fig. 2 ▸; substrate 7), amine side chains as in catecholamines (Fig. 2 ▸; substrates 8–9), as well as single and double chlorine substitutions (Fig. 2 ▸; substrates 10 and 11). Catechol oxidation by *Cl*CPO was further probed at substrate concentrations up to 0.85 *M*, without reaching saturation (Supplementary Fig. S5). Kinetic parameters could not be reliably determined due to the low catalytic rate and low apparent affinity, consistent with this activity representing a side reaction. Caffeic acid and catechin, both harbouring a catechol moiety, were not readily oxidized by either catalase (Fig. 2 ▸; substrates 12 and 13). The catecholase activity of catalases was more pronounced for chlorine-substituted substrates. In a potential ET-mediated oxidation pathway involving the haem iron, halogen substitution may enhance catechol oxidation by modulating substrate redox potential and stabilizing oxidized radical intermediates (semiquinone species), thereby lowering the overall energetic barrier (Panda *et al.*, 2025 ▸). For all substrates that catalases could oxidize, *Tt*PPO exhibited substantially faster rates than either, whereas *Cl*CPO and BLC showed comparable oxidative performance, suggestive of an intrinsic catecholase side activity in catalases in the absence of H_2_O_2_. Although such activity has sporadically been reported in the literature, its prevalence, physiological relevance and underlying mechanism remain largely unexplored.

### 3.4. Overall structure

**Figure 3:**
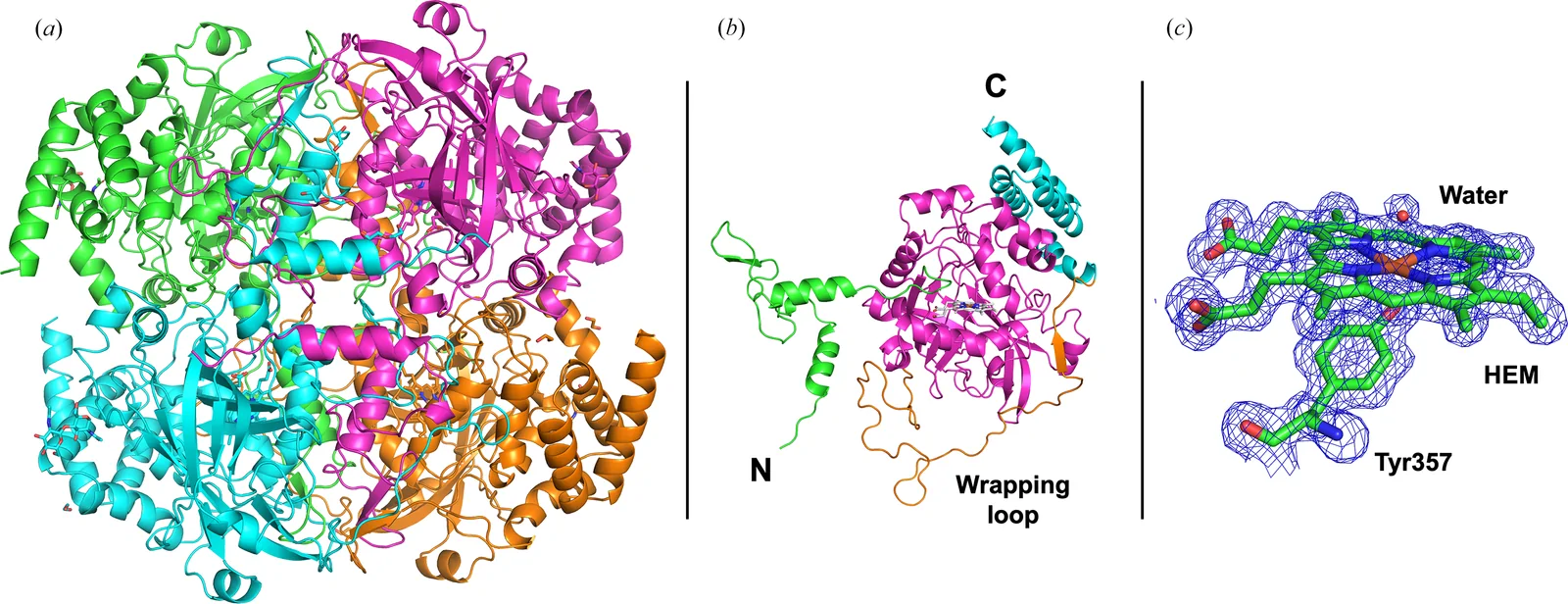
(*a*) Cartoon representation of the tetrameric form of *Cl*CPO. Each monomer is coloured separately. Haem *b* molecules and other ligands are shown as sticks. (*b*) Cartoon representation of the *Cl*CPO monomer. Termini of the protein are highlighted along with monomeric regions. (*c*) Stick representation of the haem *b*, Tyr357 and a water molecule that were modelled in the 2*F*_o_ − *F*_c_ map of molecule *H* of the *Cl*CPO crystallographic structure. The OH group of Tyr357 and a water molecule coordinate the iron ion of the porphyrin ring.

The *Cl*CPO crystal structure, determined at 1.6 Å resolution, contains two homotetramers in the asymmetric unit. There are 5122 solvent molecules in the structure and one sodium ion (Table 1 ▸). The assignment of the latter was based on the presence of close neighbours with negative charges (the carboxylates of the propionate groups of haem in monomer *H*) and its presence in the crystallization solution. Similarly to known catalases, but also indicated by *PDBePISA* analysis, the tetramer is the biologically relevant conformation (Fig. 3 ▸*a*; Krissinel & Henrick, 2007 ▸). Each monomer is comprised of 545 amino acids, involving two residues at the N-terminus and 23 residues at the C-terminus originating from the expression vector. Each monomer is composed of four domains (Fig. 3 ▸*b*): (i) the N-terminal domain (residues 4–78), which contributes to the oligomerization of the protein by interacting with neighbouring monomers and contains the distal His (His76), which is among the principal catalase catalytic residues, (ii) the β-barrel domain (residues 79–375), which comprises an antiparallel β-sheet with eight β-strands and contains the haem site, (iii) the ‘wrapping’ loop (residues 376–437), which also contributes to the oligomerization of the protein and is rich *in Pro residues, namely Pro373, Pro377, Pro403, Pro416 and Pro422, and (iv) the C-terminal domain (residues 437–505), with Asp387 being a Ramachandran plot outlier in all monomers, similarly to Asp389 and Asp400 of human (PDB entry 1dgh; Putnam *et al.*, 2000 ▸) and CATPO (PDB entry 5zz1; Yuzugullu Karakus *et al.*, 2018 ▸) catalases, respectively. *Cl*CPO belongs to the small-size subunit catalases (SSCs), as it lacks the characteristic C-terminal extension present in large-size subunit catalases (LSCs). The latter generally remain active at very high H_2_O_2_ concentrations, while SSCs are typically inhibited by substrate concentrations above 0.3–0.5 *M* (Chelikani *et al.*, 2005 ▸). The distances between the haem irons of each monomer are approximately 31 Å for the *P*-related subunits, 35 Å for the *R*-related subunits and 45 Å for the *Q*-related subunits (definition of twofold axes according to Murthy *et al.*, 1981 ▸).

**Figure 4:**
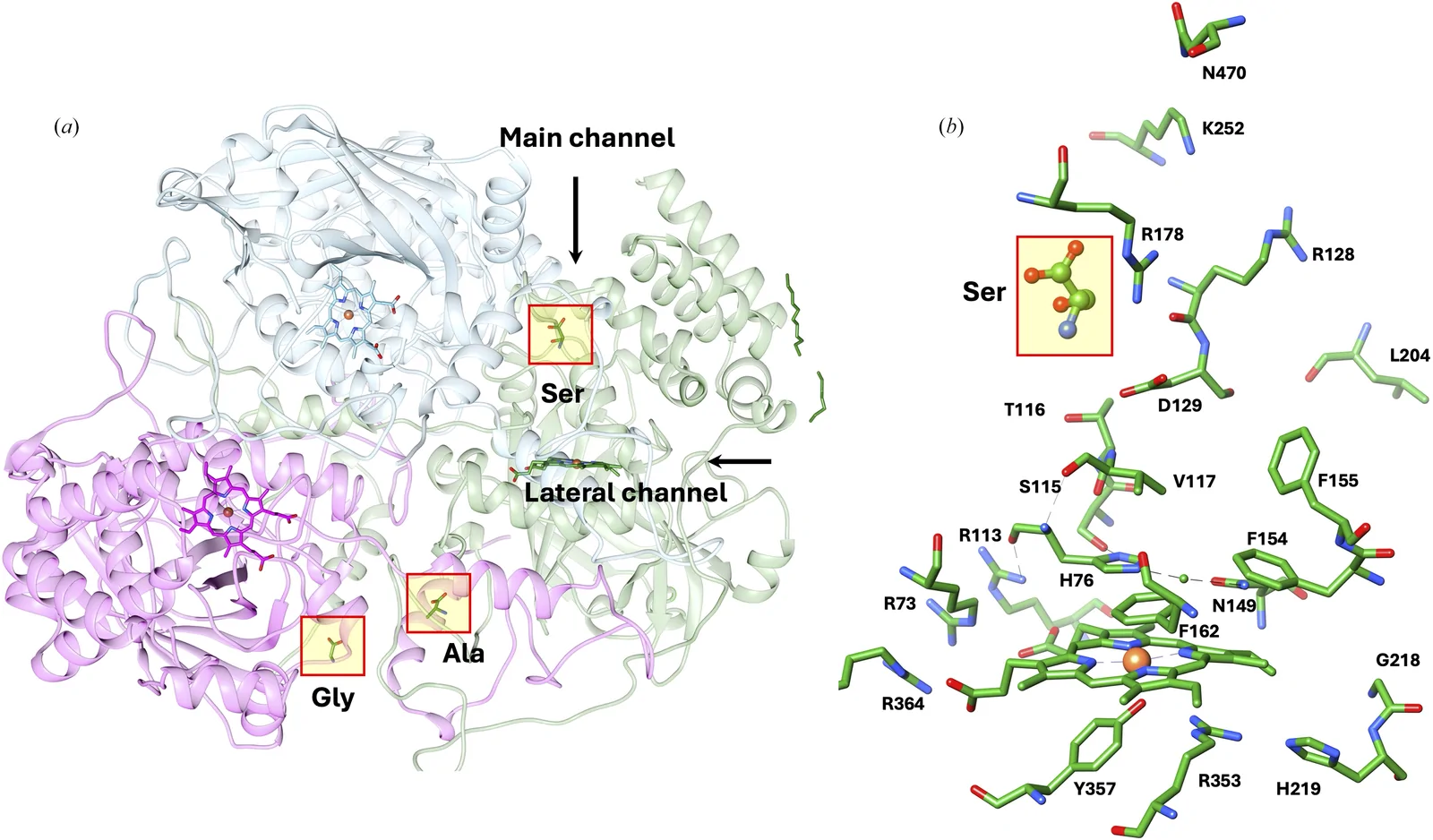
(*a*) Cartoon representation of three of the four monomers of *Cl*CPO tetramer, highlighting three serendipitously bound amino-acid residues. (*b*) Close-up view of the main channel of *Cl*CPO, showing the bound Ser residue.

Despite soaking *Cl*CPO crystals with 2HY, no electron density corresponding to ligand binding was observed. Instead, additional amino-acid residues, likely originating from the crystallization solution, were identified in the electron density. Specifically, in monomer *A* a Ser residue is modelled adjacent to Asp129, an Ala near Gln396 and Arg386 and a Gly in proximity to His5 (Fig. 4 ▸*a*). A second Gly residue is modelled in chain *F* near Asn258. Regarding the other monomers, at the position corresponding to the Ala site, *i.e.* next to Glu396, an ethane-1,2-diol (EDO) molecule is modelled in chain *D*. Ser found 16.2 Å away from the Fe atom of the haem in monomer *A* is located at the entrance to the tunnel leading to the haem active site: the so-called main catalase channel (see below).

**Figure 5:**
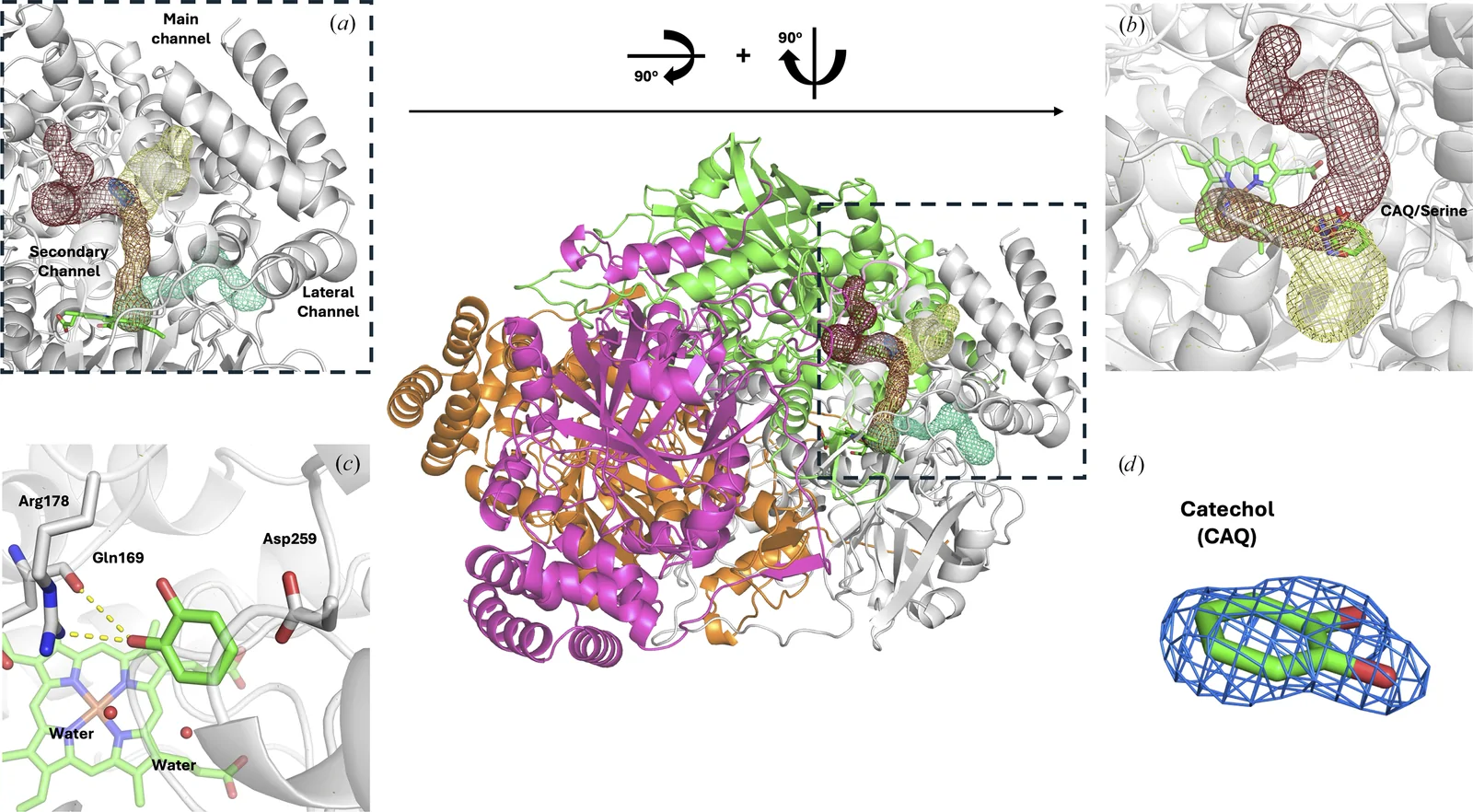
Overall view of the *Cl*CPO tetramer highlighting the channels that connect the haem active site to the solvent environment. (*a*) Detailed view of the channels shown as a mesh with different colours. (*b*) Top view of the main and secondary channels shown as a mesh. The experimentally observed Ser and CAQ positions are located at the junction of the two channels. (*c*) CAQ bound at the juncture of the main with the secondary channel shown in stick representation. Hydrogen bonds of the CAQ moiety to surrounding residues are shown as yellow dashed lines. (*d*) CAQ view in stick representation modelled within a 2*F*_o_ − *F*_c_ map shown as a blue mesh.

Catalases feature several channels which contribute to the selection of hydrogen peroxide over water, and whose dynamic nature cannot be revealed by crystallographic studies. *CAVER *3.0 analysis of the *Cl*CPO structure for the identification of tunnels that are related to the Fe atom of the haem *b* identified three relevant pathways: the main channel, a lateral channel and another secondary channel that branches from the main channel. The residues forming the channels, along with the bottleneck radius, length, curvature and overall throughput of the channels, are presented in Supplementary Table S1. The main channel includes the essential His76, Val117 and Asp129 (Asp135 in *St*CATPO), with their side chains situated 4, 8 and 12 Å from the haem iron, respectively (Fig. 4 ▸*b*). Previous studies have demonstrated the importance of this His residue for the function of the enzyme, while the side chain of the Val residue has been shown to induce narrowing of the channel leading to the haem, thus restricting access to molecules larger than H_2_O and H_2_O_2_ (Yuzugullu Karakus, 2020 ▸). The main channel is suggested to be divided into two parts: one towards the haem iron that is of hydrophobic nature, and another wider and more hydrophilic towards the enzyme surface. Also, the opening of the tunnel has been related to catalase reactivity, with wider-tunnel catalases exhibiting higher peroxidase activity, being capable of hosting larger substrates. The crystal structure of *Cl*CPO revealed a Ser molecule at the entrance of the channel, indicative of the potential to host molecules larger than H_2_O_2_ (Fig. 4 ▸*b*). The residue at the entrance to the lateral channel has been associated with catalase efficiency, specifically the bulkier the side chain, the more active the enzyme, presumably due to restricted efflux of H_2_O_2_ (Jha *et al.*, 2011 ▸). *Cl*CPO features a Gly residue (Gly218) at this position, resulting in a much wider entrance to the lateral channel compared with *St*CATPO (Val228) and human catalase (Ser217). The nature of this residue has also been related to haem orientation, with small and hydrophilic amino acids being related to AS-rIII and larger and hydrophobic ones to AS-rIV; however, this is not a strict and unbreakable rule (Jha *et al.*, 2011 ▸). The *Cl*CPO structure also reveals a secondary channel that partially overlaps with the path of the main channel but has a distinct entrance (Fig. 5 ▸*a*). As discussed in Section 3.8, docking simulations suggest a potential role for this channel in ligand binding, including the binding of phenolic substrates oxidized by *Cl*CPO.

No NADPH molecule is observed in the structure of *Cl*CPO. NADPH binding has been associated with the occurrence of two positively charged residues (Arg203 and His305 in human erythrocyte catalase; HEC) that interact with NADPH phosphate groups (Hansberg *et al.*, 2012 ▸). *Cl*CPO features Leu204 in the position corresponding to Arg203 in HEC (Fig. 4 ▸*b*), while the corresponding residue in *St*CATPO is His214. Regarding the second residue, *Cl*CPO has a shorter loop configuration in the region 293–309 compared with HEC and *St*CATPO, and features no amino acid in the location corresponding to His305 in HEC and Glu316 in *St*CATPO.

### 3.5. Active site

The active site contains a haem *b* cofactor, with the pyrrole ring III (AS-rIII) stacked against the essential His76 on the distal side of the haem, consistent with the arrangement observed in clade 3 catalases such as HEC (Ko *et al.*, 2000 ▸). Tyr357 serves as the fifth ligand to the haem iron, while electron density consistent with a water molecule coordinating the iron is observed in one of the four molecules in each homotetramer (chains *C* and *H*; Fig. 3 ▸*c*). The haem iron lies approximately 16.0 Å away from the Ser/phenolic binding site. At the pentacoordinated Fe atoms, the four N atoms of the porphyrin ring lie at a mean distance of 2.04 Å, while the phenolate oxygen from the proximal Tyr357 is at a distance of 1.97 Å (monomer *A*). The equivalent distance in the hexacoordinated Fe atoms is similar, *i.e.* 2.04 and 1.95 Å (monomer *C*), while the coordinating water molecule is at a distance of 2.03 Å from the Fe atom. The hydroxyl group of Tyr357 also interacts with N atoms NE and NH2 of Arg353. Unlike catalases such as *Neurospora crassa* CAT-I, in which this Tyr is covalently linked to a vicinal cysteine and therefore cannot act as an electron donor (Díaz *et al.*, 2004 ▸), the corresponding tyrosine in *Cl*CPO is not covalently bound to another residue and appears capable of participating in electron transfer. Nevertheless, *Cl*CPO, like *N. crassa* CAT-I, is not inhibited by H_2_O_2_, suggesting that compound II formation is prevented through an alternative mechanism. One possibility is that this Tyr donates electrons to neighbouring amino-acid residues rather than directly to the haem, thereby bypassing the pathway leading to compound II accumulation (Hansberg *et al.*, 2012 ▸). Asn149 and His76 are situated on the distal side of the haem and are connected via a water molecule (Fig. 4 ▸*b*). The NE2 atom of His76 forms hydrogen-bond interactions with a water molecule in all monomers, as well as with the water molecule acting as the sixth ligand to the haem iron in two of the eight monomers. Atom ND1 of His76 interacts with the side-chain hydroxyl group of Ser115 and the main-chain carbonyl O atom of Thr116. His76 is further stabilized through hydrogen-bond interactions of its main-chain carbonyl group with Arg113 and of its main-chain amide with the main-chain carbonyl group of Val117. Phe162 and Phe154 are also located at the haem side, contributing to the hydrophobic nature of the lower part of the channel leading to the Fe atom.

### 3.6. CAQ–*Cl*CPO

The crystal structure of *Cl*CPO complexed with catechol (CAQ–*Cl*CPO) was determined to 1.95 Å resolution. The structure was solved in the triclinic space group *P*1, which is similar to the apo *Cl*CPO structure, and the asymmetric unit contains two homotetramers (Table 1 ▸). Structural alignment of the catechol-bound homotetramer with the Ser-bound homotetramer indicates minor discrepancies between the two structures (r.m.s.d. of 0.244 Å^2^). A CAQ molecule was modelled in the main channel, 15.93 Å away from haem *b* of molecule *A* (Fig. 5 ▸). Interestingly, the experimentally observed position of catechol is similar to that of the Ser molecule found in the apo *Cl*CPO structure (Section 3.4; Figs. 4 ▸ and 5 ▸*b*). The orientation of the CAQ moiety is assisted by two hydrogen bonds formed by one of the O atoms of catechol: one to the NH_2_ group of Arg178 and another to the main-chain carbonyl O atom of Gln169 (Fig. 5 ▸*c*).

### 3.7. Comparison with *St*CATPO

**Figure 6:**
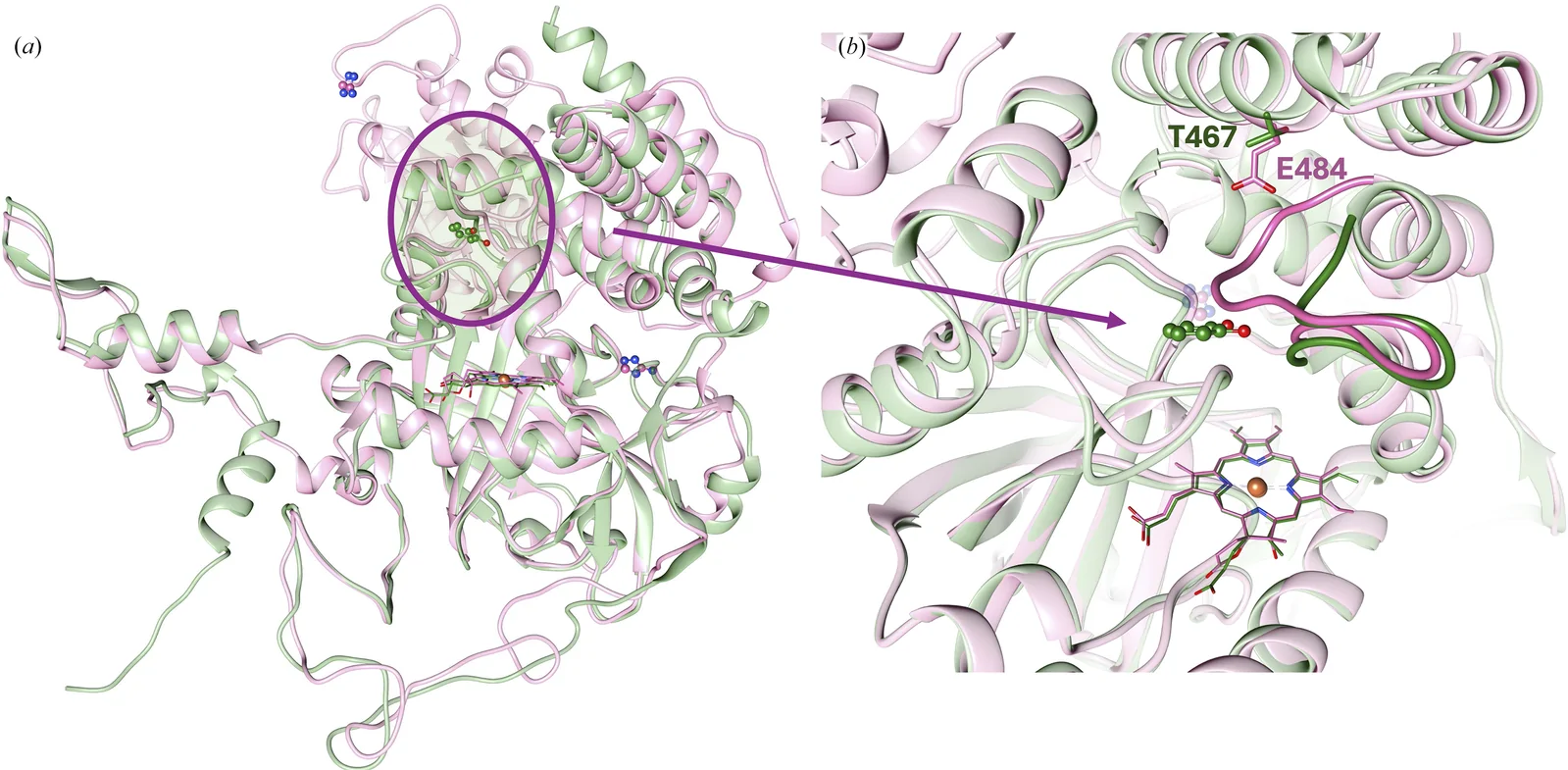
(*a*) CAQ–*Cl*CPO (molecule *A*; green) superimposed on 3TR–*St*CATPO (PDB entry 5zz1; molecule *A*; pink). (*b*) Enlarged view of the elongated loop of *St*CATPO at the entrance of the main channel.

*St*CATPO is also a bifunctional catalase capable of oxidizing small alcohols in the absence of H_2_O_2_. It features a haem *d*, in contrast to *Cl*CPO which features a haem *b*. Crystallographic data revealed the binding of the catalase inhibitor 3TR at the end of a lateral channel leading away from the haem, as well as a second binding site at the dimer interface of the homotetramer (PDB entry 5zz1; Fig. 6 ▸). The first position corresponds to the NADPH binding site in mammalian catalase (PDB entry 1dgh), and the authors suggest that a C-terminal extension (residues 533–537) in *St*CATPO is prohibitive for NADPH binding. This extension features Val536, which when replaced by Trp results in a 40% decrease in phenol oxidase activity. The corresponding region in *Cl*CPO is accessible to any potential binder, as it lacks this C-terminal extension.

The experimentally observed Ser and CAQ positions in *Cl*CPO do not correspond to the ligand-binding positions reported for *St*CATPO. In contrast, Ser and CAQ are located at the entrance of the vertical channel leading to the haem site. It should be noted that the loop located on top of the bound catechol is longer in *St*CATPO (residues 176–190) compared with *Cl*CPO (residues 170–179), with the elongated part in *St*CATPO overlapping with the bound ligands. Also, residue Glu484, which is considered to formulate the entrance of the main channel, is Thr467 in *Cl*CPO (Fig. 6 ▸*b*; Supplementary Table S2).

### 3.8. *In silico* structural analyses

Due to unsuccessful attempts to obtain crystal structures of *Cl*CPO complexed with additional oxidizable substrates, molecular-docking simulations were performed to identify plausible ligand-binding regions and poses, using the crystallographically observed catechol-binding site as a reference. Four ligands were selected for docking simulations: three phenolic substrates that are oxidized by *Cl*CPO (Section 3.3), namely 4CH (4-chlorocatechol), 35D (3,5-dichlorocatechol) and 2HY (2,3-dihydroxybiphenyl), together with PCB29 (1,2,4-trichloro-5-phenylbenzene), which was included because of its relevance to the previously proposed involvement of *Cl*CPO in PCB-associated processes. The cell for docking simulations was configured to include all three channels that are associated with ligand transport to the haem site. Rigid docking simulation was originally performed keeping all protein atoms fixed for *Cl*CPO. All generated ligand poses positioned the ligands at the entrance of the main channel. The second step of the docking-simulation strategy involved flexible docking simulations, allowing free movement of the side chains of all residues that form the main channel. Docking simulations for 4CH and 35D did not yield any results with the ligand binding deeper in the main channel. Therefore, the final strategy employed was flexible docking simulations allowing only the side chains at the entrance of the main channel to be flexible. These residues were Trp180, Asn470, Arg178, Lys252, Ser123, Asp248, Arg128, Leu200, Phe183, Gln169, Ile166, Phe197, Thr467, Lys170, Arg121 and Leu201, located in molecule *A* that forms the main channel. Additionally, two more residues from molecule *C* of the tetrameric structure (Arg69 and Asp259) that participate in the formation of the entrance of the main channel were also set free.

**Figure 7:**
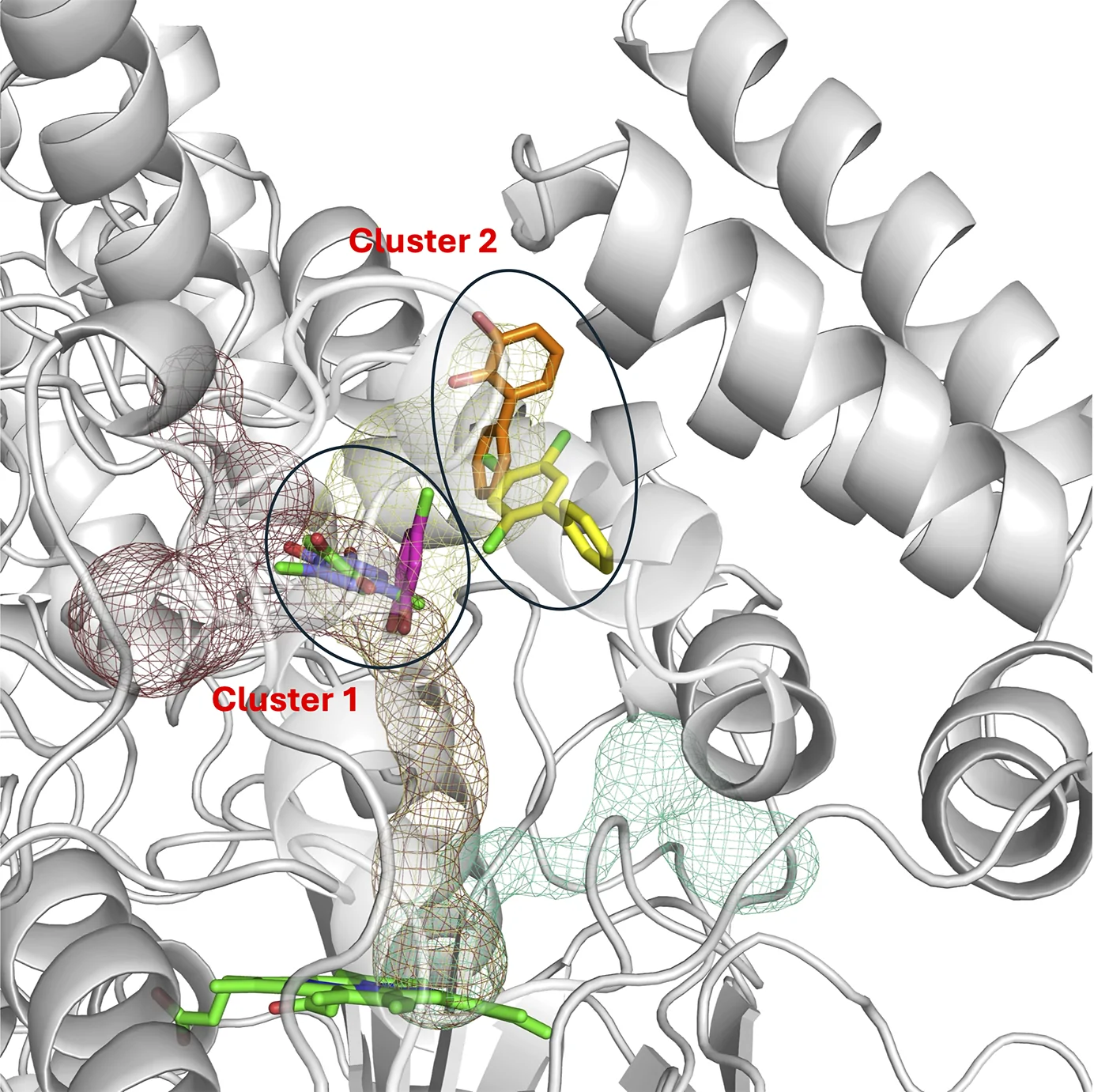
Predominant ligand-binding regions by docking simulations of 4-chloro­catechol (4CH, magenta), 3,5-dichlorocatechol (35D, purple), 2,3-di­hydroxybiphenyl (2HY, orange) and 1,2,4-trichloro-5-phenylbenzene (PCB29, yellow). The catechol position (CAQ, green) corresponds to the experimentally observed binding mode in the CAQ–*Cl*CPO crystal structure and is shown for comparison. In contrast, the positions of 4CH, 35D, 2HY and PCB29 are computationally predicted docking poses. Ligands are shown as sticks, grouped into two clusters; channels are shown in mesh representation.

Docking simulations of the four mentioned ligands resulted in several similar docking poses. Those with the highest calculated binding energy (kcal mol^−1^) and dissociation constant (*K*_d_) are presented in Fig. 7 ▸ and Supplementary Table S3. Two major areas are identified as putative ligand-binding sites. Monophenolic compounds (4CH and 35D) appear to bind at the junction of the main and the secondary channel, at the position in which the CAQ moiety was modelled in the CAQ–*Cl*CPO structure, around 16.0 Å away from the iron ion of the haem active site (Cluster 1; Fig. 7 ▸). On the contrary, bulkier biphenolic compounds (2HY and PCB29) appear to bind at the entrance to the main channel which is located around 21.0 Å away from the iron ion of the haem site (Cluster 2; Fig. 7 ▸). Specific distances of the centre of mass of docked ligands to the porphyrin iron are listed in Supplementary Table S5.

**Figure 8:**
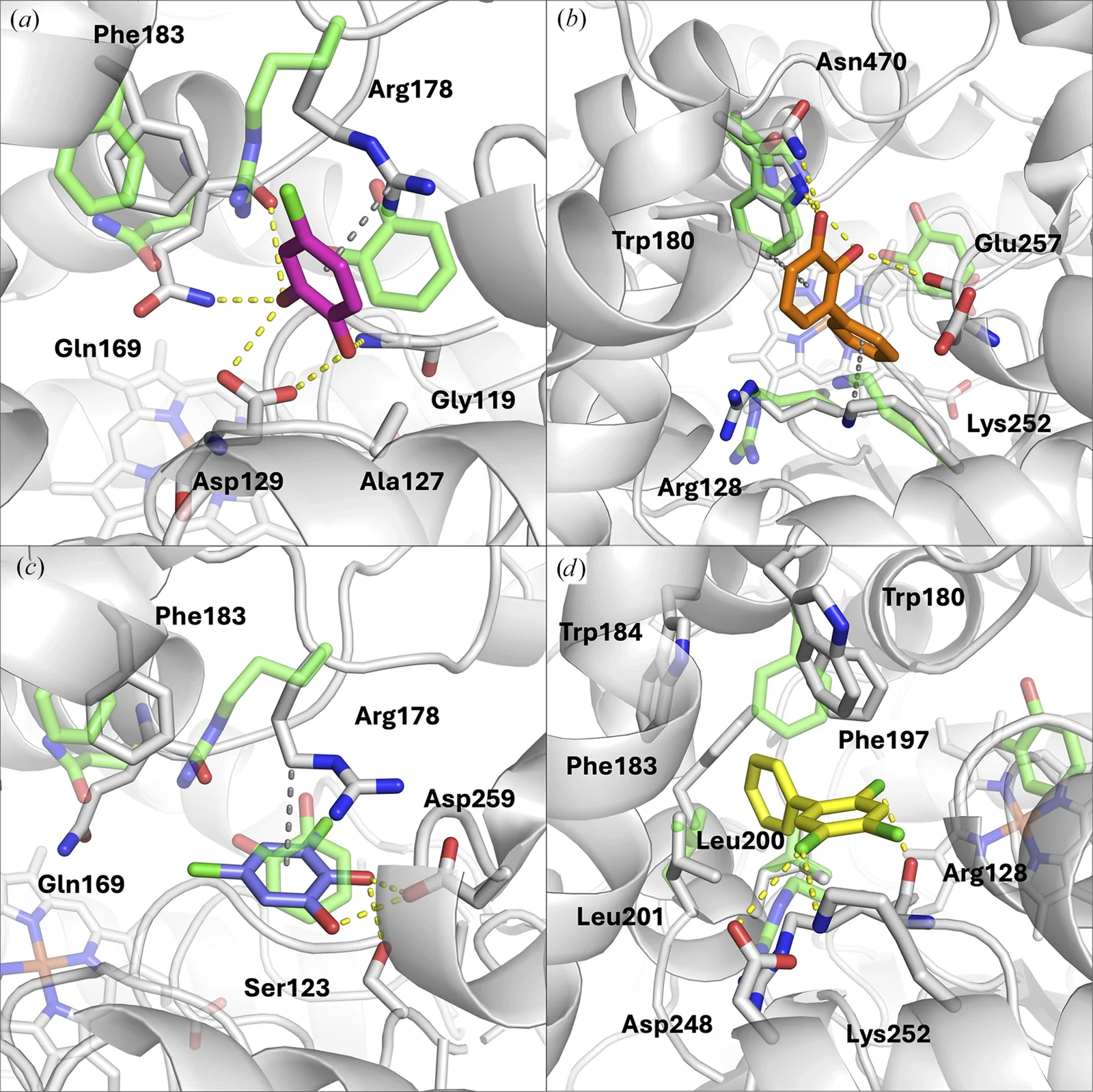
Stick representation of predicted *Cl*CPO ligand-binding regions and poses derived from docking simulations with (*a*) 4CH (magenta sticks), (*b*) 2HY (orange sticks), (*c*) 35D (blue sticks and (*d*) PCB29 (yellow sticks). Hydrogen bonds are shown as yellow dashed lines and π–π interactions as grey dashed lines. For comparison, the crystal structure of *Cl*CPO in complex with catechol is shown as green sticks.

Analysis of the predicted interactions between the docked ligands and the protein provides information on the characteristics of the candidate binding regions. The predicted docking pose of 4CH places the ligand within hydrogen-bonding distance of Asp129 and Gln169 (Fig. 8 ▸*a*). Notably, compared with the CAQ–*Cl*CPO structure there is major deviation of the side chains of residues Phe183, Gln169 and Arg178. Interestingly, the different predicted conformations of Arg178 are consistent with a possible role for this residue in modulating access to the secondary channel (Supplementary Fig. S6). For 35D, the ligand is positioned within hydrogen-bonding distance of residues Asp259, Gln169, Ser123 and Gly118 (Fig. 8 ▸*c*). Similar to 4CH, residues Arg178, Phe183 and Gln169 show deviations from the original conformation. However, the conformation of Arg178 appears to facilitate the open state of the secondary channel. The predicted docking pose of the biphenolic ligand 2HY places it at the entrance to the main channel, with potential hydrogen-bonding inter­actions involving Glu257 and Trp180 and predicted hydrophobic interactions involving Trp180, Lys252 and Arg148 (Fig. 8 ▸*b*). The predicted docking pose of PCB29 is associated with conformational changes in several hydrophobic residues relative to the CAQ–*Cl*CPO structure, resulting in a hydrophobic pocket that accommodates one of the aromatic rings of PCB29. This pocket is formed by residues Leu200, Leu201, Phe197, Trp180, Phe183, Trp184 and Thr467 (Fig. 8 ▸*d*). Overall, the docking results suggest two plausible ligand-binding regions within *Cl*CPO: a hydrophobic pocket at the entrance of the main channel and a second region near Arg178 at the junction of the main and secondary channels. These predicted binding regions may contribute to ligand recognition and provide a structural framework for generating hypotheses regarding the substrate preferences observed within the phenolic compounds examined here. The docking simulations further indicate that ligands can be accommodated at the entrance to the main channel and at several positions along the secondary channel. For instance, four distinct docking poses of 35D place the ligand at different positions within the secondary channel (Supplementary Fig. S7). These observations are consistent with a possible role of the two channels in ligand accommodation and transfer; however, docking alone does not establish their functional involvement in substrate oxidation. Such reactivity may be facilitated by extended EHT pathways within the protein matrix, analogous to other long-range redox processes described in haem enzymes (Winkler & Gray, 2014 ▸; Gray & Winkler, 2010 ▸). These pathways could enable the oxidation of bulky, aromatic substrates without direct coordination to the catalytic haem iron, potentially aided by mediator-based electron shuttling. However, molecular-dynamics simulations and complementary experimental approaches would be required to further assess ligand movement through these channels and their possible contribution to catalysis.

**Figure 9:**
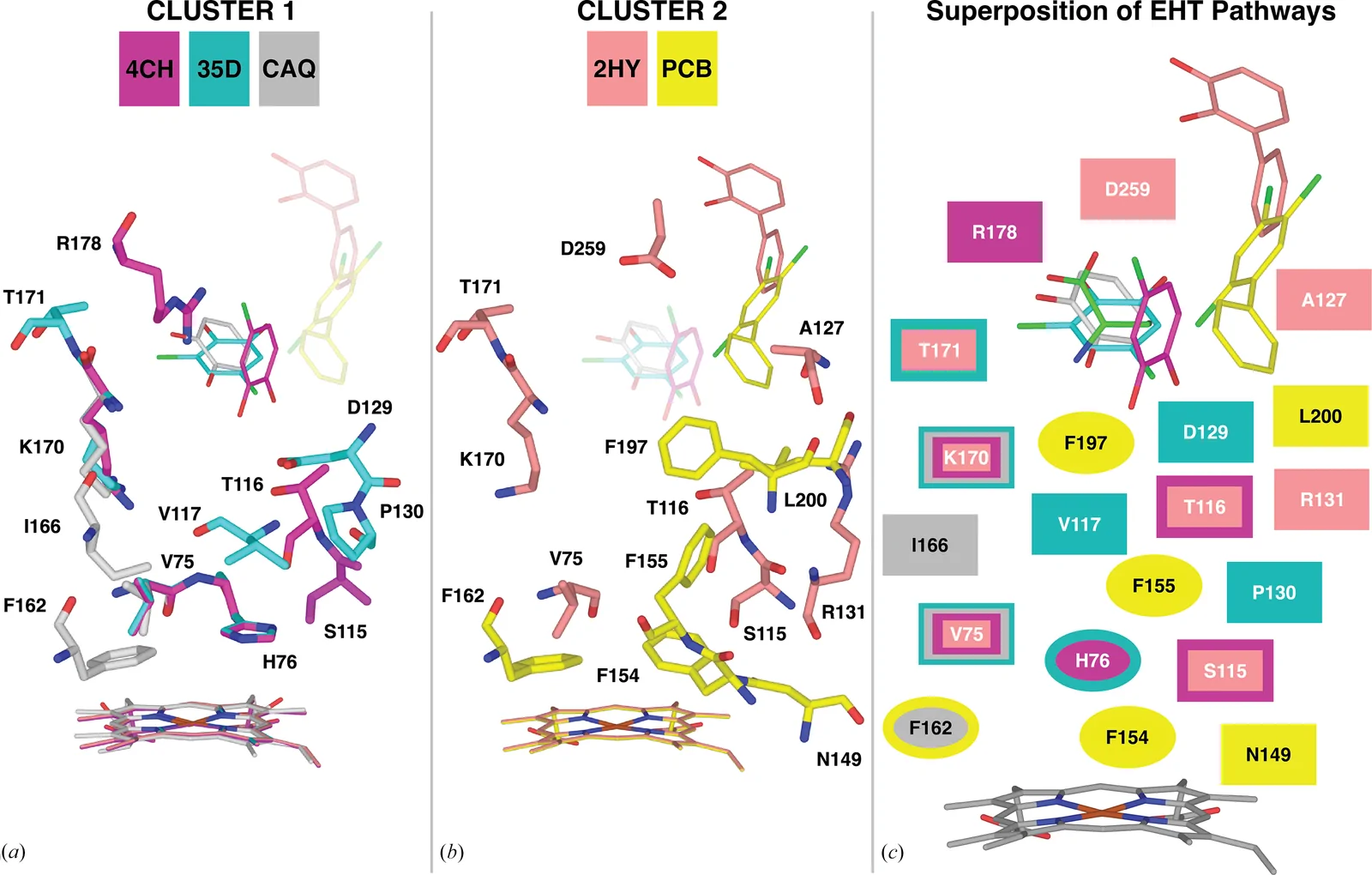
Predicted electron/hole-transfer (EHT) pathways for Cluster 1 (*a*) and Cluster 2 (*b*) of *Cl*CPO, generated computationally using *eMap* 2.0. For CAQ, the experimentally determined coordinates from the CAQ–*Cl*CPO crystal structure were used, whereas the coordinates of 4CH, 35D, 2HY and PCB29 were derived from docking simulations. Chains *A* of all structures were superposed using the *PyMOL* super function (r.m.s.d. 0.186–0.238 Å). Haem cofactors, ligands and residues predicted to participate in EHT are shown as sticks. (*c*) Superposition of predicted EHT pathways. A single representative haem cofactor and all five ligands are shown as sticks. Residues participating in EHT are depicted schematically at their centre-of-mass coordinates (ovals, aromatic residues; rectangles, non-aromatic residues). Colours correspond to the ligand-bound structures shown in (*a*) and (*b*). The three highest ranked predicted pathways per structure are shown; all ten predicted pathways per structure are provided in Supplementary Table S4. 4CH, 4-chlorocatechol; 35D, 3,5-dichlorocatechol; CAQ, catechol; 2HY, dihydroxyphenyl; PCB, PCB29 (1,2,4-trichloro-5-phenylbenzene).

To explore possible electron-transfer routes between the ligand-binding regions and haem *b*, putative EHT pathways were predicted *in silico* (Tazhigulov *et al.*, 2019 ▸). For each docked substrate, ten putative EHT pathways were identified and ranked according to their overall score. All ten pathways are summarized in Supplementary Table S4, while the three highest ranking pathways for each structure are presented in Fig. 9 ▸. For Cluster 1 ligands (4CH, 35D and CAQ) the predicted EHT routes involve residues Val75, His76, Ser115, Thr116, Val117, Asp129, Pro130, Phe162, Ile166, Lys170, Thr171 and Arg178. Residues Val75, His76 and Ser115 are predicted to constitute the terminal part of these pathways, and could potentially provide relay steps connecting the ligand-binding region to the haem site (Fig. 9 ▸*a*). For Cluster 2 ligands (2HY and PCB29) the predicted pathways involve residues Val75, Ser115, Thr116, Ala127, Arg131, Asn149, Phe154, Phe155, Phe162, Lys170, Thr171, Phe197, Leu200 and Asp259 (Fig. 9 ▸*b*). Notably, superposition of all predicted EHT pathways reveals a considerable degree of conservation among the residues supporting EHT for Cluster 1 ligands, whereas the predicted pathways associated with Cluster 2 ligands show greater ligand-specific variation (Fig. 9 ▸*c*). In particular, the EHT pathways associated with PCB29 are supported by four Phe residues that do not participate in any of the other predicted pathways, while the pathways associated with 2HY share five relay residues with those predicted for Cluster 1 ligands. Among these, Val75 and Lys170 are present in all analysed pathways and therefore emerge as recurrent elements of the predicted electron-transfer network, irrespective of the ligand-binding subsite. Collectively, the predicted EHT pathways reveal recurrent pathway elements within *Cl*CPO, with several residues shared among routes originating from different ligand-binding subsites. These common features suggest that distinct binding regions may connect to the haem cofactor through partially overlapping predicted electron-transfer pathways. Importantly, the EHT pathways identified here represent computational predictions and should be considered as plausible mechanistic hypotheses rather than experimentally established electron-transfer routes. Their functional involvement in *Cl*CPO-mediated substrate oxidation has not been directly validated, for example through site-directed mutagenesis of the predicted relay residues or complementary biochemical approaches. Further experimental investigation will therefore be required to establish whether these predicted pathways contribute to electron transfer during catalysis.

## 4. Conclusions

The findings presented here advance our current understanding of the structural basis underlying the secondary phenol oxidase activity of catalases. Crystallographic studies identified a catechol-binding region, while molecular-docking simulations suggested an additional candidate binding region capable of accommodating bulkier ligands. Both subsites are located within the main channel leading to the haem iron, making them plausible candidate binding regions that may contribute to the phenol oxidase activity of *Cl*CPO. However, their direct involvement as sites of substrate oxidation has not been experimentally established. Notably, the experimentally identified catechol-binding region differs from that reported for *St*CATPO, the only structurally characterized catalase with phenol oxidase activity (Yuzugullu *et al.*, 2013 ▸), as well as from the putative binding site proposed for mammalian catalase in the β-barrel haem-bearing domain (Vetrano *et al.*, 2005 ▸). These differences may reflect distinct substrate specificities and catalytic roles among bifunctional catalases. In both experimentally determined structures, the bound ligands are found at a distance from the haem iron, indicative of catalysis taking place via electron transfer. However, the phenol-binding site identified in *Sc*CATPO is located at the beginning of a C-terminal region that is absent in *Cl*CPO. This region corresponds to the NADPH-binding domain found in many large-subunit catalases and may have been repurposed in *Sc*CATPO to accommodate phenolic substrates. In contrast, the binding site observed in *Cl*CPO is in a structurally distinct region, namely the main channel, that is narrower in *Sc*CATPO. Since these enzymes possess several channels connecting the protein surface to the haem active site and serving as pathways for substrate access and product release, their dimensions and physicochemical properties determine selectivity. It can thus be suggested that different structural solutions can support phenol oxidase activity within the catalase scaffold.

Collectively, these findings support a working mechanistic model in which phenolic substrates may undergo oxidation while bound at discrete subsites within the main haem channel, potentially involving electron transfer to the haem cofactor. However, the proposed electron-transfer pathways are based on computational predictions and have not yet been experimentally validated; consequently, alternative mechanisms, including transient direct interaction of the substrates with the haem iron, cannot be excluded. Although previous transcriptomic data implicated *Cl*CPO in the response of *Cladosporium* sp. to PCB29 exposure, the present biochemical data do not demonstrate direct PCB29 transformation by the enzyme. Its possible physiological involvement in pollutant degradation therefore remains unresolved. This emerging view of phenol oxidase activity in catalases, together with the apparent diversity of ligand-binding regions among bifunctional catalases, provides a structural framework that may inform future efforts to engineer catalases with tailored phenol oxidase activities for environmental bioremediation and other industrial biocatalytic applications.

## Supplementary Material

Supplementary Figures and Tables. DOI: 10.1107/S2059798326009848/nz5023sup1.pdf

PDB reference: bifunctional catalase–phenol oxidase from a marine-derived *Cladosporium* species, 9hxt

PDB reference: complex with catechol, 9rwy

## Figures and Tables

**Table 1:** Crystallographic data and refinement statistics for *Cl*CPO structures Values in parentheses are for the outer shell.

	Apo *Cl*CPO (PDB entry 9hxt)	CAQ–*Cl*CPO (PDB entry 9rwy)
Diffraction source	P13, PETRA III	P13, PETRA III
Wavelength (Å)	0.9763	0.9763
Temperature (K)	100	100
Detector	Dectris EIGER X 16M	Dectris EIGER X 16M
Crystal-to-detector distance (mm)	178.579	234.013
Rotation range per image (°)	0.05	0.2
Total rotation range (°)	360	360
Exposure time per image (s)	0.01	0.01
Space group	*P*1	*P*1
*a*, *b*, *c* (Å)	92.55, 92.66, 169.23	91.87, 92.08, 168.81
α, β, γ (°)	83.31, 78.18, 60.33	83.00, 77.94, 60.10
Mosaicity (°)	0.24	0.27
Resolution range (Å)	82.94–1.60 (1.63–1.60)	82.52–1.95 (1.98–1.95)
Total No. of reflections	2132491 (95896)	1190376 (56222)
No. of unique reflections	577755 (26249)	310143 (14508)
Completeness (%)	91.6 (83.6)	90.7 (85.8)
Half-set correlation CC_1/2_	0.992 (0.549)	0.990 (0.387)
Multiplicity	3.7 (3.7)	3.8 (3.9)
〈*I*/σ(*I*)〉	7.0 (1.5)	5.0 (0.8)
*R*_merge_	0.107 (0.883)	0.146 (1.468)
*R*_p.i.m._	0.066 (0.534)	0.087 (0.861)
Overall *B* factor from Wilson plot (Å^2^)	17.46	27.11
No. of reflections, working set	577036 (29038)	310143 (14508)
No. of reflections, test set	29071	15401
Final *R*_cryst_	0.199	0.256
Final *R*_free_	0.236	0.295
No. of non-H atoms		
Protein	33057	32694
Ion	1	—
Ligand	701	496
Water	5122	2328
R.m.s. deviations		
Bond lengths (Å)	0.0103	0.0101
Angles (°)	1.939	1.821
Average *B* factors (Å^2^)		
Protein	19.54	38.1
Ion	20.76	—
Ligand	23.63	33.91
Water	30.72	42.32
Ramachandran plot		
Most favoured (%)	96.47	96.32
Outliers (%)	0.22	0.30
Crystallization condition	10%(*w*/*v*) PEG 20K, 20%(*v*/*v*) PEG MME 550, 0.02 *M* of each amino acid, 0.1 *M* MES/imidazole pH 6.5; amino acids: sodium L-glutamate, 0.2 *M*DL-alanine, 0.2 *M* glycine, 0.2 *M*DL-lysine–HCl, 0.2 *M*DL-serine	0.12 *M* Monosaccharides (D-glucose, D-mannose, D-galactose, L-fucose, D-xylose, *N*-acetyl-D-glucosamine), 0.1 *M* imidazole/MES pH 8.5, 30.00%(*v*/*v*) PEG 500 MME, 30.00%(*v*/*v*) PEG 20K

## Data Availability

All data supporting the findings of this study are available within the article and its supporting information. The atomic coordinates and structure factors for the crystal structures determined in this study have been deposited in the PDB under accession codes 9hxt and 9rwy.
