# Supplementary material for: Beyond monofunctionality: structural perspectives on phenol oxidase side activity in a catalase
Source: Acta Crystallogr D Struct Biol. 2026 Oct 8;82(Pt 11):1224–39. doi: 10.1107/S2059798326009848 (PMC13717827; doi:10.1107/S2059798326009848)
Supplement: Supplementary file 1 [file d-82-01224-sup1.pdf]

STRUCTURAL BIOLOGY

Volume 82 (2026)

Supporting information for article:

Beyond monofunctionality: structural perspectives on phenol oxidase side activity in a catalase

Christina Ferousi, Christos Kosinas, Odyssefs Ioannis Pantelakis, Efstratios Nikolaivits, Evangelos Topakas and Maria Dimarogona

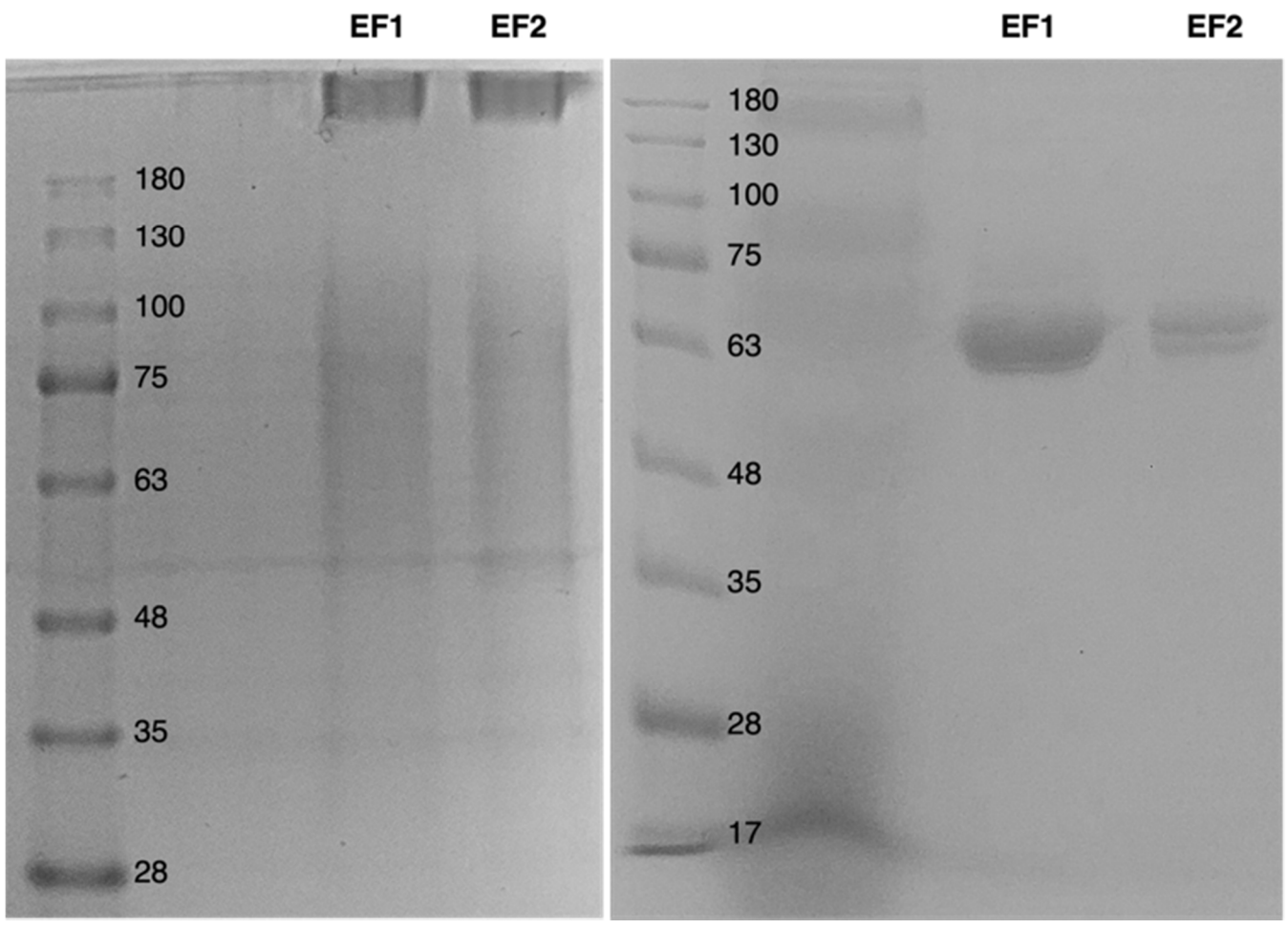

Figure S1 Purified *Cl*CPO resolved by SDS-PAGE. Elution fractions (EF; 20 μg per lane) resulting from 100 mM imidazole application to the immobilized metal affinity chromatography were resolved by reducing 10% SDS-PAGE and visualized by Coomassie staining along with molecular weight markers (kDa) as indicated. *Left:* as isolated; *Right:* after treatment with endoglycosidase H.

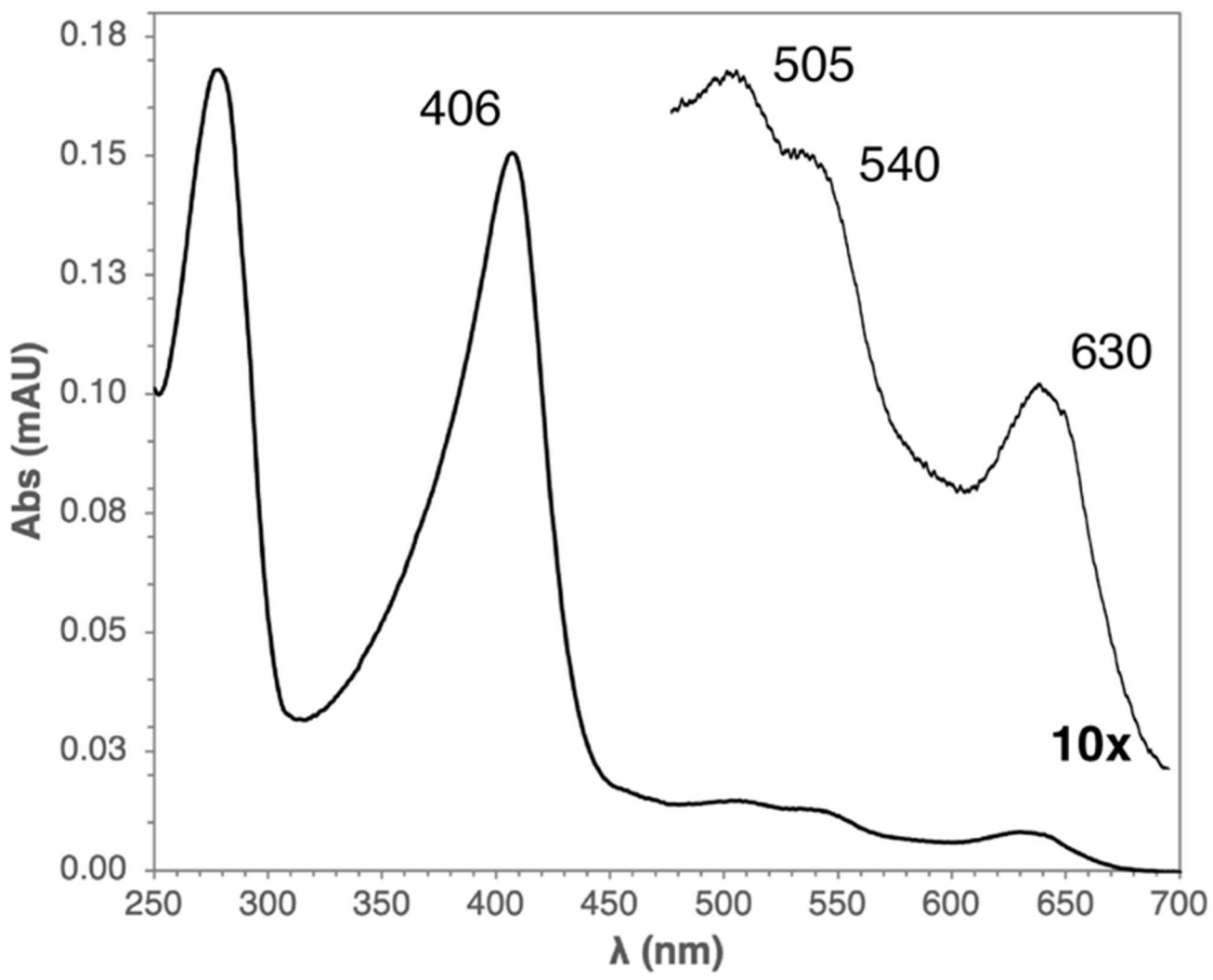

Figure S2 Electronic absorption spectra of as isolated (fully oxidized) *Cl*CPO. The inset shows a 10-fold magnification of the 480 to 700 nm region.

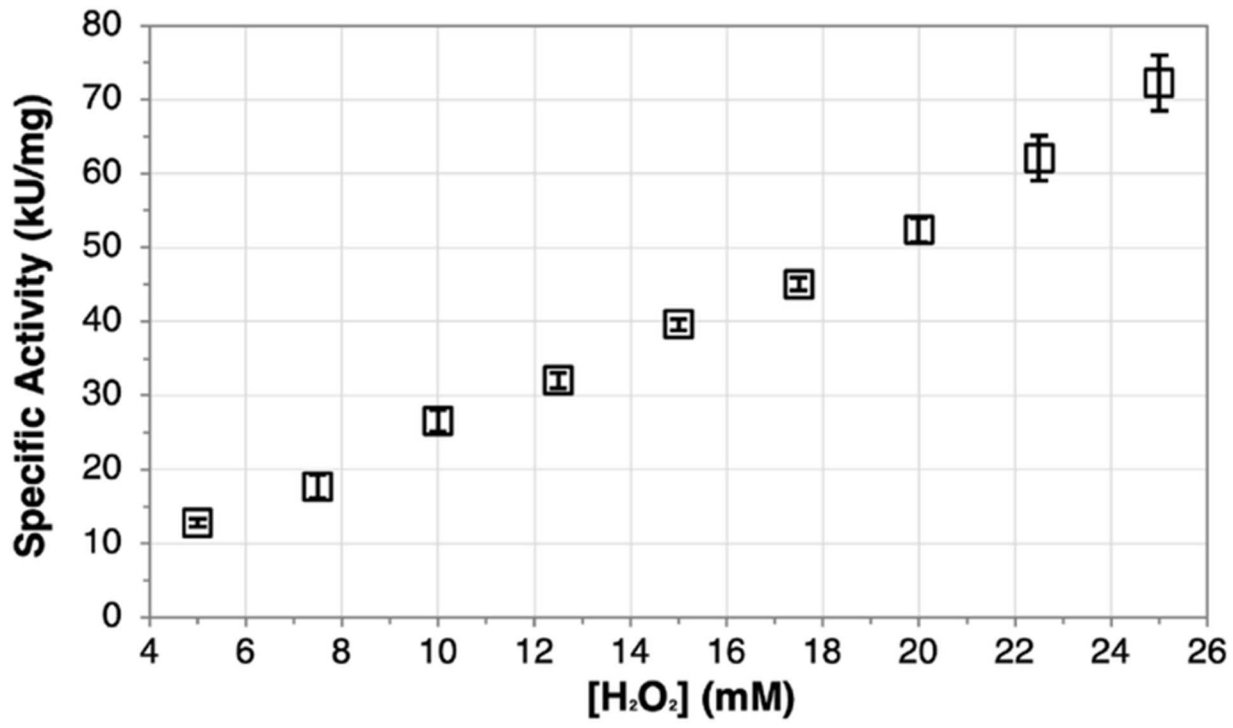

Figure S3 Catalase activity of *Cl*CPO (1.2 nM) as a function of H_2O_2 concentration. Reactions were carried out in 50 mM potassium phosphate buffer pH 8.0 at room temperature (RT). H_2O_2 dismutation was followed by the decrease in absorbance at 240 nm (ε_{240} = 39.4 M^{-1} cm^{-1}) during the first 20 seconds of the reaction. Values represent the mean of three measurements; error bars indicate standard deviation.

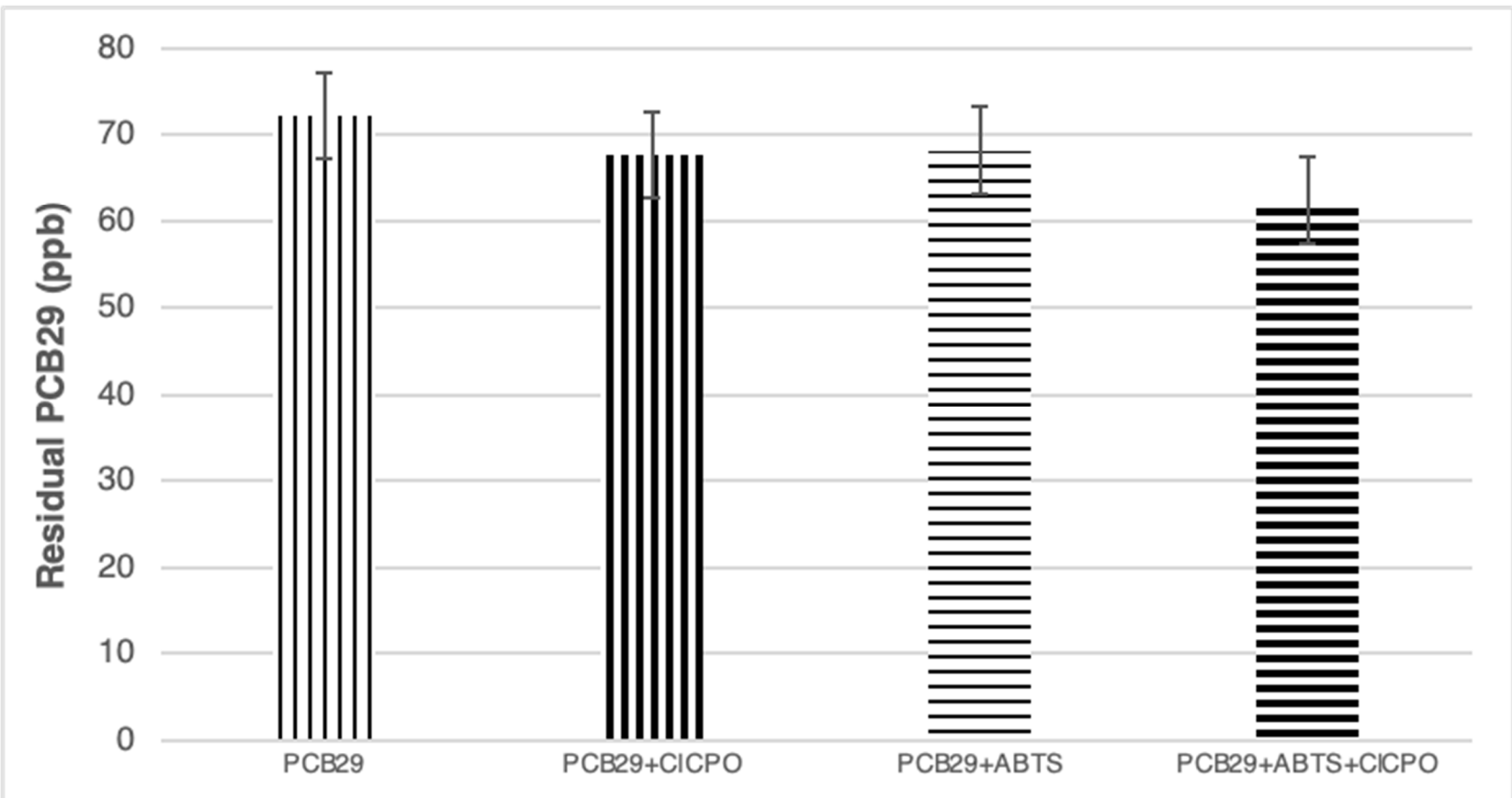

Figure S4 *Cl*CPO-dependent transformation of PCB29. Residual PCB29 concentrations (ppb) after incubation are shown for control and reaction mixtures containing PCB29 alone, PCB29 with *Cl*CPO, PCB29 with ABTS, and PCB29 with both ABTS and *Cl*CPO. Statistical analysis showed that these differences were not significant relative to the corresponding controls. Reactions were performed in 0.1 M potassium phosphate buffer (pH 7.0) with 1 µM PCB29, 1 µM ABTS (where indicated), and 5 µM *Cl*CPO at 35 °C for 24 h, followed by extraction and GC–ECD analysis.

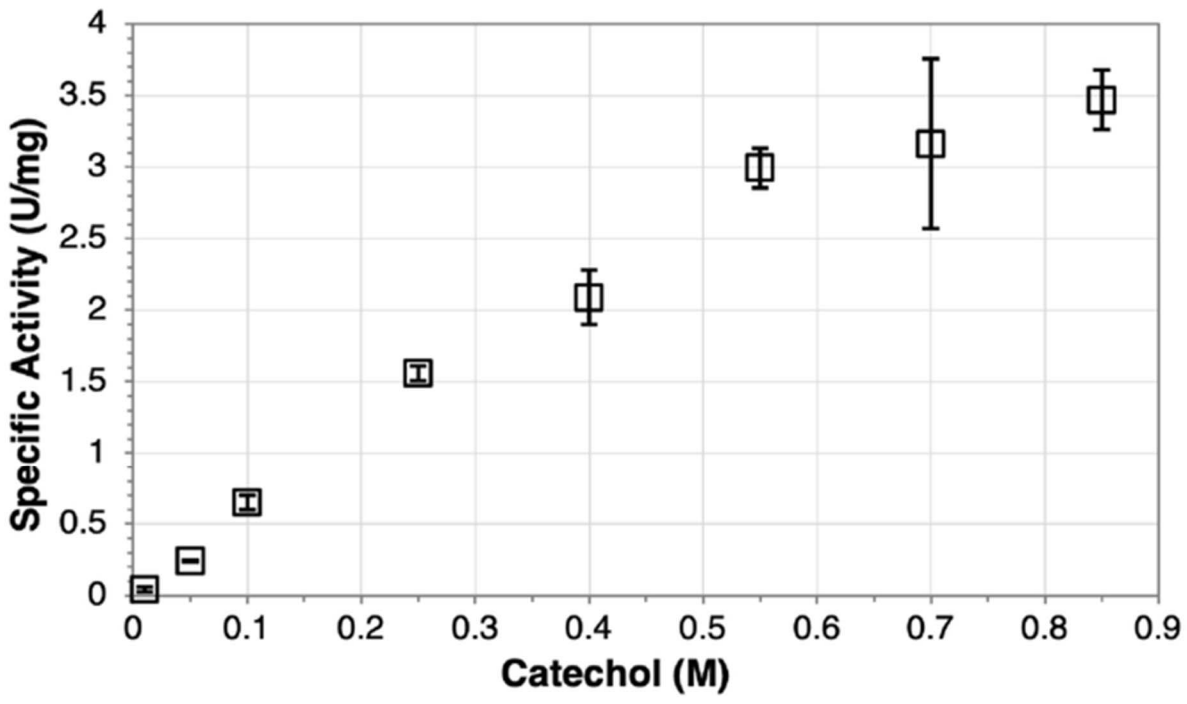

Figure S5 Catecholase activity of *Cl*CPO (1.5 µM) as a function of catechol concentration. Reactions were carried out in 0.1 M potassium phosphate buffer pH 7.0 at 35°C. Catechol oxidation was followed by the decrease in absorbance at 420 nm (ε_{420} = of 3,450 M^{-1} cm^{-1}) during the first 2 minutes of the reaction. Values represent the mean of two measurements; error bars indicate standard deviation.

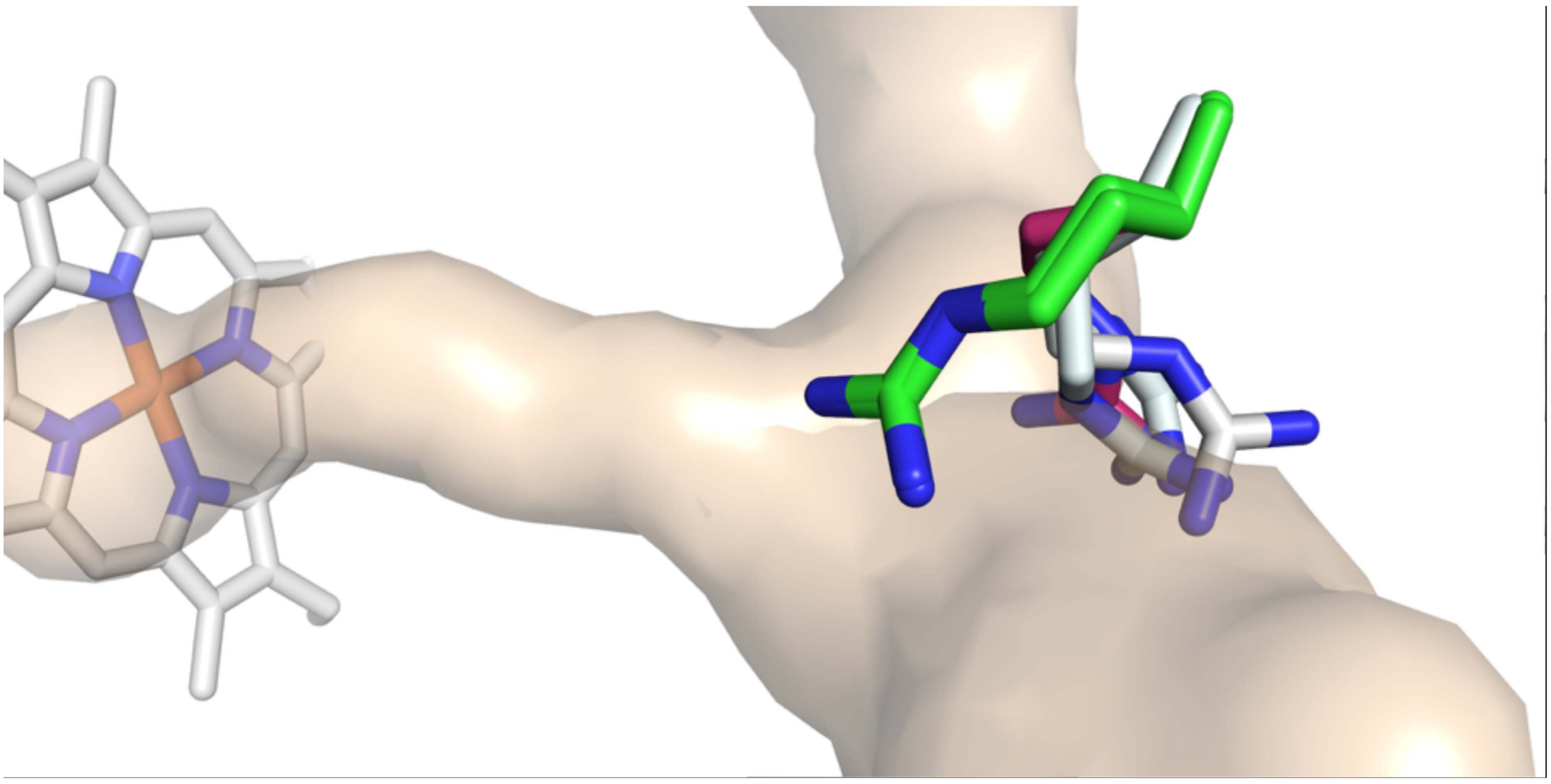

Figure S6 Superposition of different conformations of Arg178, illustrating its proposed role in modulating access to the secondary channel. Arg178 in the experimentally determined CAQ-*Cl*CPO structure is shown in green, whereas Arg178 conformations derived from docking simulations with 35D, 2HY, PCB29, and 4CH are shown in grey or magenta as indicated. Main and secondary channels are shown as wheat surface and the haem *b* as grey sticks.

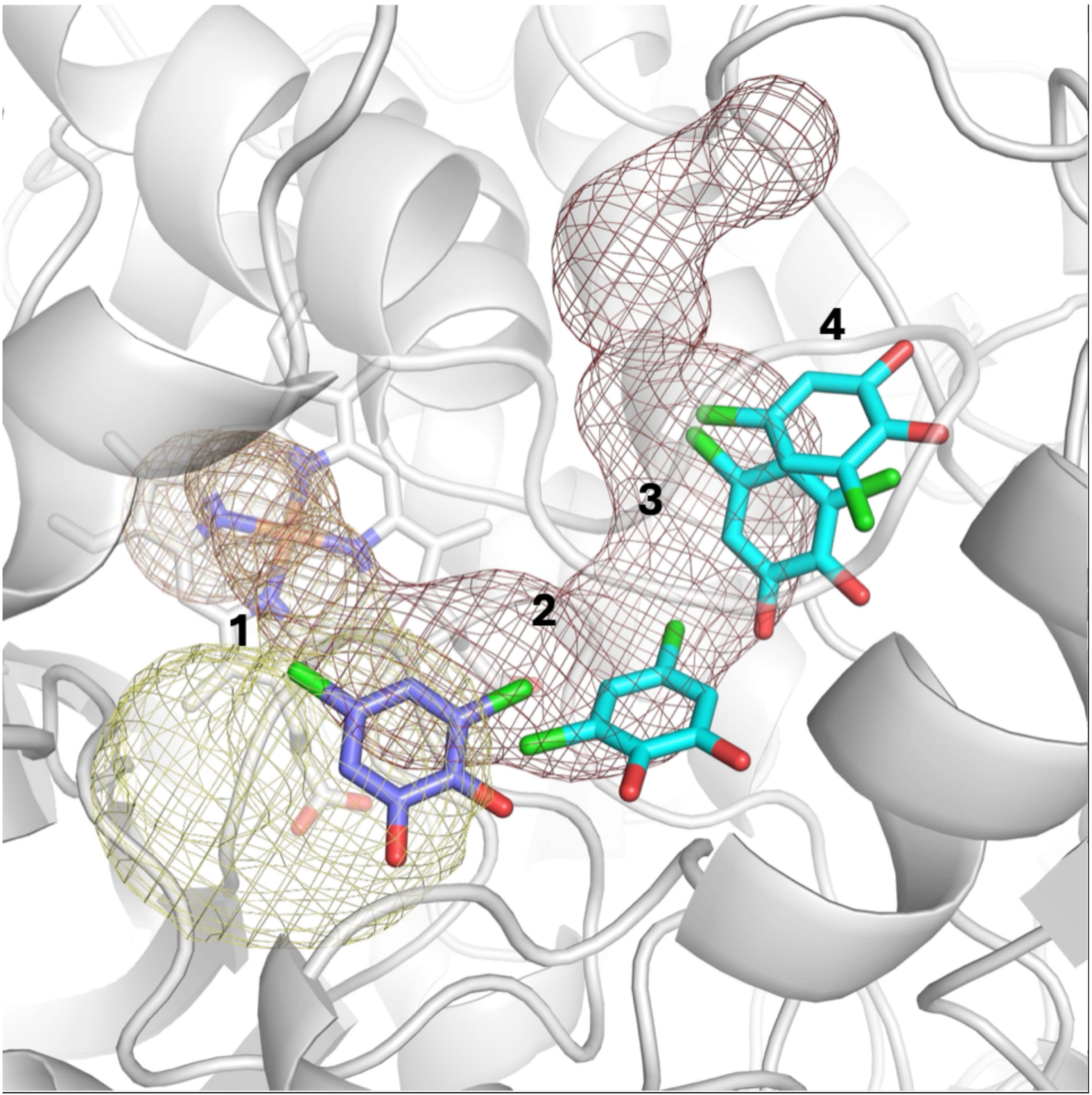

Figure S7 Four predicted docking poses of 35D within the secondary channel as predicted by docking simulations. The protein is illustrated as cartoon, the ligand is shown in sticks, the channels in mesh representation, while the haem *b* molecule is shown as sticks.

Table S1 Tunnel characteristics as estimated by the CAVER 3.0 PyMOL plugin. Bottleneck radius is an estimation of the narrowest radius identified in the tunnel, while throughput reflects the predicted ability of a tunnel to transport small molecules.

Channel	Bottleneck radius (Å)	Length (Å)	Curvature (Å)	Throughput	Residues forming the channel
Main channel	1.27	29.56	1.28	0.45	**Chain A:** Thr116, Val117, Gly118, Gly119, Ala120, Ser123, Ala124, Thr126, Ala127, Arg128, Asp129, Pro130, Val147, Phe148, Asn149, Phe154, Phe155, Phe162, Phe165, Ile166, Gln169, Lys170, Thr171, Arg178, Trp180, Phe183, Thr206, His219, Asp248, Lys252, Ala255, Val349, Arg353, Tyr357, Gly466, Thr467, Asn470<br>**Chain C:** Ala255, Gly256, Glu257, Asn258, Asp259, Ala260
Lateral channel	0.92	23.76	1.43	0.32	**Chain A:** Val75, His76, Val117, Pro130, Arg131, Val147, Asn149, Asn150, Thr151, Pro152, Phe154, Phe155, Phe162, Ile166, Gln196, Arg199, Leu200, Ser202, Leu204, Pro207, Met213, His214, Trp216, His219, Val243, Asn245, Val349, Arg353, Tyr357, Ser115, Thr116, Asp129, Gly132, Phe133, Phe148, Val153, Leu201, Gly205, Thr206, His212, Gly215

Secondary channel	1.14	44.72	1.88	0.22	**Chain A:** Glu72, Val75, His76, Val117, Ala120, Ser123, Ala127, Asp129, Pro130, Val147, Asn149, Phe154, Phe155, Phe162, Phe165, Ile166, His167, Thr168, Gln169, Lys170, Thr171, Asp172, Pro173, Ala174, Asn176, Arg178, Asp179, Thr182, Phe183, His219, Val349, Arg353, Tyr357, Val74, Gly118, Gly119, Arg121, Arg128, Phe148, Ala177<br>**Chain C:** Phe65, Glu68, Arg69, Arg78, Arg121, Asp259, His325, Gly122

Table S2 Comparison of amino acid residues of the haem environment and the NADPH binding pocket in *Cl*CPO and *St*CATPO (PDB ID: 4AUM).

Feature	*Cl*CPO	CATPO
Proximal Haem Side	Y357	Y369
	R353	R365
	H219	H229
	D347	D359
Distal Haem Side	H76	H82
	N149	N155
	S115	S121
	T116	T122
Haem Propionate Groups	R73	R79
	R113	R119
	R364	R376
Haem Vinyl Group (entrance of lateral channel)	G218	V228
Main Haem Channel	V75	V81
	V117	V123
	P130	V136
	F154	F160
	F155	F161
	F162	F168
	I166	I172
	D129	D135
	Q169	V175
	G119	G125
	R128	R134
	T467	E484
	A127	A133

NADPH Binding Pocket	L195	H205
	S202	S212
	L204	H214
	H214	D214
	E238	K248
	W304	I314
	N306	Y318
	Q443	Q461

Table S3 Results of molecular docking simulations with the highest calculated binding energies (Figure 7). 4-chlorocatechol: 4CH; 3,5-dichlorocatechol: 35D; 2,3-dihydroxybiphenyl: 2HY; 1,2,4-trichloro-5-phenylbenzene: PCB29.

Cluster No	Ligand	Binding energy ((kcal mol−1)	K_d (mM)	Contact Receptor Residues
1	4CH	5.072	0.191000	**Chain A**: Thr116, Gly118, Gly119, Ser123, Ala127, Arg128, Asp129, Gln169, Arg178, Trp180, Phe183
1	35D	5.520	0.089000	**Chain A**: Gly118, Gly119, Ala120, Ser123, Ala124, Ala127, Arg128, Asp129, Gln169, Lys170, Thr171, Arg178, Phe183<br>**Chain C**: Arg78, Asp259
2	2HY	5.545	0.086000	**Chain A**: Ala127, Arg128, Trp180, Asp248, Lys252, Gly466, Thr467, Asn470<br>**Chain C**: Gly256, Glu257
2	PCB29	9.147	0.000197	**Chain A**: Ala127, Arg128, Trp180, Phe183, Trp184, Phe197, Leu200, Leu201, Asp248, Lys252, Thr467

Table S4 Predicted EHT pathways calculated using eMap for all ligand-bound *Cl*CPO structures. For CAQ, the experimentally determined coordinates from the CAQ-ClCPO crystal structure were used, whereas the coordinates of 4CH, 35D, 2HY, and PCB29 were derived from docking simulations. Ten ranked EHT pathways are listed for each ligand-bound structure. Pathways are reported as ordered residue sequences connecting the haem cofactor to the ligand, with residues given in the predicted sequential order of EHT. Pathways are ranked according to the eMap penalty score, with lower scores corresponding to more favourable pathways. 4CH: 4-chlorocatechol; 35D: 3,5-dichlorocatechol; CAQ: catechol; 2HY: dihydroxybiphenyl; PCB: PCB29 (1,2,4-trichloro-5-phenylbenzene).

Ligand	Rank	Pathway Residues (Haem to Ligand)					eMap Score*	No of Steps
4CH	1	S115	T116	—	—	—	15.39	3
4CH	2	V75	K170	R178	—	—	16.57	4
4CH	3	H76	S115	T116	—	—	17.05	4
4CH	4	S115	T116	D129	—	—	17.73	4
4CH	5	N149	S115	T116	—	—	19.68	4
4CH	6	S115	T116	A127	—	—	20.15	4
4CH	7	S134	S115	T116	—	—	20.21	4
4CH	8	V75	H76	S115	T116	—	21.46	5
4CH	9	V75	K170	A77	T116	—	23.43	5
4CH	10	S115	R113	A77	T116	—	24.44	5
35D	1	H76	V117	D129	—	—	15.88	4
35D	2	H76	V117	P130	D129	—	18.28	5
35D	3	V75	K170	T171	—	—	18.84	4
35D	4	S115	T116	D129	—	—	19.15	4
35D	5	S115	T116	S123	—	—	20.07	4
35D	6	S115	T116	A127	—	—	20.15	4
35D	7	V75	H76	V117	D129	—	20.31	5
35D	8	V75	K170	T171	R178	—	21.49	5
35D	9	H76	S115	T116	S123	—	21.73	5
35D	10	V75	K170	E72	A120	—	21.86	5

CAQ	1	V75	K170	—	—	—	13.1	3
CAQ	2	F162	I166	K170	—	—	15.7	4
CAQ	3	V75	I166	K170	—	—	15.9	4
CAQ	4	H76	V117	D129	—	—	16.2	4
CAQ	5	H76	V75	K170	—	—	16.6	4
CAQ	6	V75	V117	D129	—	—	17.6	4
CAQ	7	V74	V75	K170	—	—	17.7	4
CAQ	8	V75	K170	T171	—	—	18.0	4
CAQ	9	V74	V75	V117	D129	—	22.3	5
CAQ	10	V74	F162	T116	K170	—	22.4	5
2HY	1	S115	T116	R131	A127	—	25.13	5
2HY	2	V75	K170	T171	D259	—	26.24	5
2HY	3	S115	T116	S123	D259	—	28.30	5
2HY	4	V75	I166	K170	T171	D259	28.89	6
2HY	5	V75	K170	T171	D259	A127	29.87	6
2HY	6	S115	T116	S123	R178	W180	29.90	6
2HY	7	V75	K170	T171	N176	D259	30.99	6
2HY	8	S115	T116	R78	S123	D259	32.70	6
2HY	9	V74	R69	R121	N176	D259	32.85	6
2HY	10	S115	T116	R131	A127	D259	32.97	6
PCB	1	N149	F154	F155	L200	—	17.3	5
PCB	2	N149	F154	F155	F197	—	17.4	5
PCB	3	F162	F154	F155	L200	—	17.9	5
PCB	4	F162	F154	F155	F197	—	18.0	5
PCB	5	H76	V117	Q169	L200	—	18.1	5
PCB	6	H76	V117	P130	L200	—	18.2	5
PCB	7	H76	V117	Q169	F183	—	18.3	5

PCB	8	H76	V117	D129	L200	—	20.0	5
PCB	9	S115	T116	D129	L200	—	23.2	5
PCB	10	S115	T116	R131	A127	—	26.5	5

* The number of steps corresponds to the total number of nodes connecting the haem cofactor and the ligand. Empty pathway positions are indicated by an em dash (—). eMap scores are reported as calculated by the software without further normalization.

Table S5 Estimated straight-line distances of the center of mass of the docked ligands to the iron of the heam active site of molecule A.

Ligand	Distance (Å)
4CH	15.12
35D	15.52
2HY	23.71
PCB	19.75
